# Investigation of how gate residues in the main channel affect the catalytic activity of *Scytalidium thermophilum* catalase

**DOI:** 10.1107/S2059798323011063

**Published:** 2024-01-24

**Authors:** Yonca Yuzugullu Karakus, Gunce Goc, Melis Zengin Karatas, Sinem Balci Unver, Briony A. Yorke, Arwen R. Pearson

**Affiliations:** a Department of Biology, Kocaeli University, Kabaoglu, Kocaeli, Izmit 41001, Türkiye; b School of Chemistry, Faculty of Engineering and Physical Sciences, University of Leeds, Leeds LS2 9JT, United Kingdom; c The Hamburg Centre for Ultrafast Imaging, Institute for Nanostructure and Solid State Physics, HARBOR, Universitat Hamburg, 22761 Hamburg, Germany; University of Oxford, United Kingdom

**Keywords:** catalases, phenol oxidases, main channel gate residues, oxidoreductases, *Scytalidium thermophilum*, catalase variants

## Abstract

Catalase is an antioxidant enzyme that breaks down hydrogen peroxide (H_2_O_2_) into molecular oxygen and water. In all monofunctional catalases the pathway that H_2_O_2_ takes to the catalytic centre is via the ‘main channel’. However, the structure of this channel differs in large-subunit and small-subunit catalases. In large-subunit catalases the channel is 15 Å longer and consists of two distinct parts, including a hydrophobic lower region near the heme and a hydrophilic upper region where multiple H_2_O_2_ routes are possible. Conserved glutamic acid and threonine residues are located near the intersection of these two regions. Mutations of these two residues in the *Scytalidium thermophilum* catalase had no significant effect on catalase activity. However, the secondary phenol oxidase activity was markedly altered, with *k*_cat_ and *k*_cat_/*K*_m_ values that were significantly increased in the five variants E484A, E484I, T188D, T188I and T188F. These variants also showed a lower affinity for inhibitors of oxidase activity than the wild-type enzyme and a higher affinity for phenolic substrates. Oxidation of heme *b* to heme *d* did not occur in most of the studied variants. Structural changes in solvent-chain integrity and channel architecture were also observed. In summary, modification of the main-channel gate glutamic acid and threonine residues has a greater influence on the secondary activity of the catalase enzyme, and the oxidation of heme *b* to heme *d* is predominantly inhibited by their conversion to aliphatic and aromatic residues.

## Abstract 2

*Scytalidium thermophilum* produces a catalase enzyme that is capable of oxidizing *o*-diphenolic and some *p*-diphenolic compounds in the absence of hydrogen peroxide. To better understand the role of the main channel in phenol oxidase activity, the gate residues Glu484 and Thr188 in the upper part of the main channel were investigated in a combined kinetic, spectroscopic and structural study.

## 1. Introduction

During aerobic respiration, both prokaryotic and eukaryotic organisms consume molecular oxygen (O_2_) and form reactive oxygen species (ROS) as a byproduct of their metabolism. When ROS accumulate in large quantities in the cell, they cause oxidative stress and have a damaging effect on macromolecules such as nucleic acids, lipids and proteins. Catalases reduce oxidative stress by breaking down hydrogen peroxide (H_2_O_2_) to water and molecular oxygen (Maté *et al.*, 2001 ▸).

The catalytic reaction of catalases proceeds in two distinct phases. The first stage involves the oxidation of the resting enzyme (Por–Fe^3+^) by H_2_O_2_ to a porphyrin cation radical ( 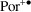 –Fe^4+^=O) (equation 1). This radical intermediate, termed compound I, is reduced by another H_2_O_2_ molecule to regenerate the enzyme in the resting state and the products H_2_O and O_2_ (equation 2). Under low H_2_O_2_ concentrations, if a suitable organic compound is present, compound I can be reduced to compound II (equation 3), which reacts with H_2_O_2_ to form the inactive compound III (equation 4), ultimately causing enzyme inhibition. For NADPH-binding catalases, it has been proposed that the enzyme is protected from the formation of compound III by NADPH, thereby preventing or rescuing the formation of compound II (Sevinc *et al.*, 1999 ▸; Putnam *et al.*, 2000 ▸; Nicholls, 2012 ▸). 
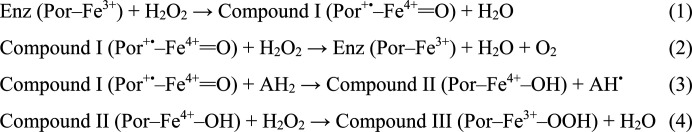
 Catalases are classified into three groups based on sequence and structural differences: the heme-containing monofunctional catalases, the heme-containing bifunctional catalase peroxidases and the manganese catalases, which contain manganese instead of heme. Although they use different mechanisms, all catalyse the cleavage of H_2_O_2_ into water and molecular oxygen (Nicholls *et al.*, 2001 ▸). The most widespread and well studied class of catalases, the monofunctional catalases, are phylogenetically divided into three distinct clades. The enzymes of clade 1 consist mainly of plant catalases, with a subgroup of bacterial origin (Díaz *et al.*, 2012 ▸). The clade 1 catalase structures so far determined all contain noncovalently bound heme *b* (ferriprotoporphyrin IX) and do not bind NADPH. Clade 2 catalases are all enzymes with large subunits (>75 kDa) and are found predominantly in bacteria and fungi. Their larger size results from an additional 50 residues at the N-terminus and a flavodoxin-like domain with 150 residues at the C-terminus. They exhibit an unusual resistance to thermal degradation, denaturants such as detergents, organic solvents and salts, and proteolytic cleavage (Chelikani *et al.*, 2004 ▸). A slightly larger number of crystal structures have been published for clade 2 enzymes, including 12 from variants of *Scytalidium thermophilum* catalase (also known as catalase–phenol oxidase or CATPO; Yuzugullu, Trinh, Fairhurst *et al.*, 2013 ▸; Yuzugullu, Trinh, Smith *et al.*, 2013 ▸; Yuzugullu Karakus *et al.*, 2018 ▸; Goc *et al.*, 2021 ▸), than for clade 1 enzymes (Díaz *et al.*, 2012 ▸). It has been shown that clade 2 enzymes predominantly possess heme *d*, which is a *cis*-hydroxyspirolactone and an oxidized derivative of heme *b* (Díaz *et al.*, 2012 ▸). Due to the extended C-terminal region reaching into the NADPH-binding site, these enzymes cannot bind NADPH. Clade 3 catalases are enzymes with small subunits (<60 kDa). They are derived from a diverse group of fungi, bacteria, archaea and animals. Many crystal structures of clade 3 catalases are available, showing that they all contain heme *b* (Díaz *et al.*, 2012 ▸). Among all clades, these are the only enzymes that bind NADPH (Chelikani *et al.*, 2004 ▸).

**Figure 1:**
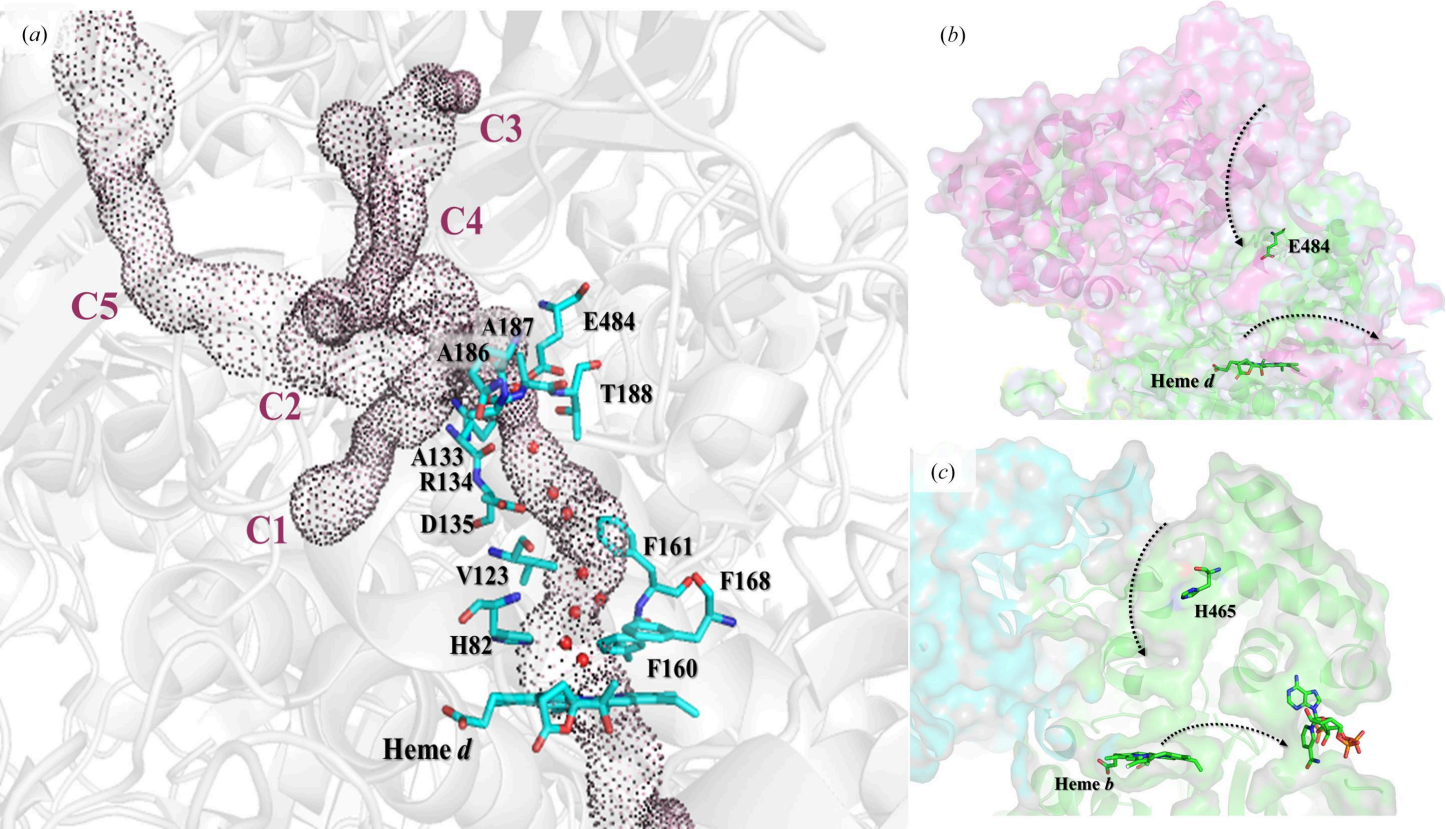
(*a*) The main-channel architecture in CATPO. The complexity of the channel above Thr188 and Glu484 is evident from its branching into C1, C2, C3, C4 and C5 channels. All channels reach the surface. The channel cavities were calculated using *Caver* (Chovancova *et al.*, 2012 ▸). (*b*, *c*) Comparison of the main and lateral channel structures leading to the active sites of the dimer of the large-subunit catalase CATPO (*b*) and the dimer of the small-subunit catalase bovine liver catalase (BLC) (*c*). Glu484 in CATPO and the corresponding residue His465 in BLC, located at the entrance of the main channel, are indicated. CATPO and BLC are oriented in the same way for ease of comparison. The figure was generated using *PyMOL* (https://www.pymol.org/).

All monofunctional catalases have a typical core structure in which a heme-containing active site is deeply buried and access to heme is provided by three channels (main, central and lateral). These channels are quite tightly arranged and only allow H_2_O_2_ and smaller molecules to pass through them (Switala & Loewen, 2002 ▸). The main channel is thus named as there is biochemical evidence that it may be the primary pathway for access of H_2_O_2_ to the active site, which is supported by molecular-dynamics studies (Kalko *et al.*, 2001 ▸). The main channels of large-subunit and small-subunit enzymes differ in length and structure; for example, in large-subunit enzymes the main channel is 15 Å longer and contains a complex multiple passage in the upper part which passes through the additional C-terminal domain of the adjacent subunit (Figs. 1 ▸ *a* and 1 ▸ *b*). A conserved glutamic acid (Glu484 in CATPO and Glu530 in *Escherichia coli* hydroperoxidase or HPII) is located at the junction between the hydrophobic lower part and the hydrophilic upper part of the channel. This elongated and more constricted channel in catalases with large subunits is thought to cause lower turnover rates (Díaz *et al.*, 2012 ▸). In contrast, in enzymes with small subunits the entrance to the main channel is an open funnel (Fig. 1 ▸ *c*), where the equivalent position to Glu484 in CATPO, where the funnel narrows, is a glutamate in *Proteus mirabilis* catalase (PMC), a histidine in bovine liver catalase (BLC), an alanine in *Micrococcus lysodeikticus* catalase (MLC), a serine in *Helicobacter pylori* catalase (HPC) and an asparagine in *Vibrio salmonicida* catalase (VSC) (Supplementary Fig. S1).

In large-subunit catalases the gate and small cavity are formed by Arg134, Glu484 (CATPO numbering) and a loop of four residues (Gln185, Ala186, Ala187 and Thr188 in CATPO). In small-subunit catalases the arginine is conserved, but they lack the loop of four amino-acid residues (Díaz *et al.*, 2004 ▸). The highly conserved four-residue loop located directly above the hydrophobic lower part of the main channel in large-subunit catalases is thought to act as a gate allowing access of H_2_O_2_ to the lower part of the main channel, while reducing water access (Díaz *et al.*, 2012 ▸). Molecular-dynamics studies of *Neurospora crassa* CAT-1 support this proposal (Domínguez *et al.*, 2010 ▸). In this regard, after soaking the crystals of the inactive H128N variant of HPII with H_2_O_2_, H_2_O_2_ was shown to be hydrogen-bonded to Glu530 (equivalent to Glu484 in CATPO) and Ser234 (equivalent to Thr188 in CATPO) (Melik-Adamyan *et al.*, 2001 ▸). This glutamate (Glu484 in CATPO) is fully conserved by the gate residues in large-subunit catalases, whereas serine is only found in half of the sequenced large-subunit catalases and is a threonine in the other half (Supplementary Table S1).

Catalase (CATPO) from the thermophilic fungus *S. thermophilum* is a clade 2 enzyme consisting of four subunits, each with a molecular weight of 80 kDa. In addition to its primary catalase activity, it also has a minor phenol oxidase activity that is capable of oxidizing *o*-diphenolic and some *p*-diphenolic compounds in the absence of hydrogen peroxide (Ögel *et al.*, 2006 ▸; Sutay Kocabas *et al.*, 2008 ▸; Yuzugullu, Trinh, Smith *et al.*, 2013 ▸). This phenol oxidase activity is oxygen-dependent and probably occurs in a similar manner to NADPH oxidation by electron transfer from the substrate (located in the likely binding site for phenolic substrates) to a high-valent iron–oxo intermediate, which is presumably formed by reaction with oxygen (Koclar Avci *et al.*, 2013 ▸; Sutay Kocabas *et al.*, 2008 ▸; Yuzugullu, Trinh, Smith *et al.*, 2013 ▸; Yuzugullu Karakus *et al.*, 2018 ▸). We have previously shown that mutations in the lower hydrophobic part of the main channel result in altered catalase and phenol oxidase activities, as well as different patterns of ordered solvent molecules in the main channel (Yuzugullu, Trinh, Fairhurst *et al.*, 2013 ▸). With the aim of better understanding the role of the main channel in phenol oxidase activity, we investigated the gate residues Glu484 and Thr188 in the upper part of the main channel in a combined kinetic, spectroscopic and structural study.

## 2. Experimental procedures

### 2.1. Materials

KOD Hot Start DNA Polymerase, 30%(*w*/*w*) hydrogen peroxide solution, catechol, pyrogallol, 4-*tert*-butylcatechol, 4-methylcatechol, 2,2′-azino-bis(3-ethylbenzthiazoline-6-sulf­onic acid) (ABTS), *p*-hydroquinone, sodium azide, sodium fluoride, 3-amino-1,2,4-triazole (3TR), potassium cyanide and salicylhydroxamic acid were purchased from Sigma and Merck.

### 2.2. Construction, expression and purification of the CATPO variants

Oligonucleotides (Table 1 ▸) with the desired mutations (E484A, E484D, E484I, T188A, T188D, T188F and T188I) were obtained from Sentegene and used to mutate the *catpo* gene in the pET-28-TEV-CATPO plasmid according to the procedure described previously (Yuzugullu, Trinh, Smith *et al.*, 2013 ▸). Mutations were confirmed by DNA sequencing (MedSantek, Türkiye) on an ABI 3730XL Sanger sequencing device (Applied Biosystems, USA) using the BigDye Terminator v3.1 Cycle Sequencing Kit (Applied Biosystems, USA). Thereafter, sequential expression and purification were performed as described previously (Yuzugullu, Trinh, Smith *et al.*, 2013 ▸; Yuzugullu, Trinh, Fairhurst *et al.*, 2013 ▸).

### 2.3. Enzyme assays

Catalase activity was measured according to the method described by Beers & Sizer (1952 ▸) with some modifications (Yuzugullu, Trinh, Smith *et al.*, 2013 ▸). One unit of catalase is defined as the amount that catalyses the degradation of 1 µmol H_2_O_2_ in 1 min in 10 m*M* H_2_O_2_ solution at pH 7.0 at 60°C. All catalase assays were performed in triplicate in 100 m*M* sodium phosphate buffer. Phenol oxidase activity was determined using catechol (420 nm, ɛ = 3450 *M* ^−1^ cm^−1^) as a substrate (Ögel *et al.*, 2006 ▸). In some assays, pyrogallol (430 nm, ɛ = 2470 *M* ^−1^ cm^−1^; Pruitt *et al.*, 1990 ▸), 4-*tert*-butylcatechol (420 nm, ɛ = 1150 *M* ^−1^ cm^−1^; Waite, 1976 ▸), 4-methylcatechol (420 nm, ɛ = 1350 *M* ^−1^ cm^−1^; Waite, 1976 ▸), *p*-hydroquinone (250 nm, ɛ = 9310 *M* ^−1^ cm^−1^; Ishigami & Yamada, 1986 ▸) and ABTS (420 nm, ɛ = 36 000 *M* ^−1^ cm^−1^; Childs & Bardsley, 1975 ▸) were used as substrates. One unit of phenol oxidase is defined as the amount that catalyses the formation of 1 nmol product per minute at 60°C. The concentration range used to calculate the kinetic parameters varied from 1 to 150 m*M* for *p*-hydroquinone, catechol, 4-methylcatechol and pyrogallol and from 0.1 to 10 m*M* for 4-*tert*-butylcatechol and ABTS. All reactions were performed in triplicate at pH 7.0, except for the ABTS oxidation assay, which was carried out in 100 m*M* acetate buffer pH 5.0.

The initial rates were used to calculate the conversion rates (*k*_cat_), *V*_max_ and *K*_m_ by fitting *v* versus [S] traces to the Michaelis–Menten equation using *SigmaPlot* 14.0 (Systat Software). Inhibition experiments were performed under the same conditions, except that the inhibitor was dissolved in reaction buffer at the indicated concentrations. The inhibition type and inhibitor constant (*K*_i_) for each inhibitor were derived from the double-reciprocal plot (Lineweaver–Burk curve; Lineweaver & Burk, 1934 ▸) using six concentrations of the catechol substrate ranging from 1 to 100 m*M*. All inhibition assays were performed in triplicate. The protein concentration was determined according to the method of Bradford (1976 ▸). All data were recorded using a temperature-controlled Cary60 spectrophotometer (Agilent).

### 2.4. Absorbance spectroscopy

The absorbance spectra of recombinant CATPO and its variants were recorded in a quartz cuvette (1 cm path length) between 250 and 750 nm. All spectra were recorded using a temperature-controlled Cary60 spectrophotometer (Agilent).

### 2.5. Crystallization and structure determination

The hanging-drop vapour-diffusion method was used to obtain crystals of the CATPO variants. The reservoir solutions consisted of 200 m*M* KCl, 10 m*M* CaCl_2_, 50 m*M* C_2_H_7_AsO_2_ with 6–16%(*v*/*v*) polyethylene glycol 400 at pH 5.0–5.6. A cryoprotective solution containing 20%(*v*/*v*) polyethylene glycol 400 was used to prevent damage when flash-cooling the crystals in liquid nitrogen (Teng, 1990 ▸). Diffraction data were collected on beamline ID29 (Proposal Nos. mx1799 and mx1849) at the European Synchrotron Radiation Facility (ESRF; de Sanctis *et al.*, 2012 ▸; McCarthy *et al.*, 2018 ▸) at 100 K (Table 2 ▸). Processing, scaling, model building and refinement of the diffraction data were performed with *XDS*, *Coot* and *REFMAC*5 in the *CCP*4 suite (Agirre *et al.*, 2023 ▸) using the native CATPO structure (PDB entry 4aum) as a starting model for *MOLREP* (Vagin & Teplyakov, 2010 ▸). The E484A, T188A and T188F variant structures were determined at 1.78, 1.40 and 1.49 Å resolution, respectively. The data were slightly anisotropic and the resolution was cut where CC_1/2_ dropped below 0.3 on the weakest axis. The structures were deposited in the Worldwide Protein Data Bank as entries 7wca, 7vn0 and 5yem, respectively. *PyMOL* (https://www.pymol.org/) and *Caver* (Chovancova *et al.*, 2012 ▸) were used to generate images.

## 3. Results and discussion

### 3.1. Kinetic and spectroscopic characterization of the Glu484 and Thr188 variants

Because of their locations, Glu484 and Thr188 are important residues in the main channel of CATPO. Therefore, we investigated the role of Glu484 and Thr188 in both the catalase and phenol oxidase activities of the CATPO enzyme by mutating them to apolar (E484A and T188A), aliphatic (E484I and T188I), acidic (E484D and T188D) and aromatic (T188F) amino acids. Soluble proteins could be expressed for all variants, indicating that protein folding was not affected by any of the mutations.

Kinetic analysis (Table 3 ▸) showed that the catalase activity changed moderately between the wild-type CATPO enzyme and its variants. When comparing the *K*_m,app_ values of CATPO and its seven variants, we found that the *K*_m,app_ values of the E484I, T188D and T188I variants were about twofold higher than those of wild-type CATPO, while the *K*_m,app_ values of the other variants were very similar to those of CATPO. Similar values or a slight increase were also observed for the turnover numbers (*k*_cat_), except for the E484A and T188F variants. The E484A and T188F variants showed a decrease in turnover by 53% and 48%, respectively. The catalytic efficiencies (*k*_cat_/*K*_m,app_), on the other hand, were reduced by 6–43% for all variants. These results show that mutations of Glu484 and Thr188, which are both located at the entrance to the main channel, affect the catalase activity by less than 45%. In agreement with our results, a previous study of the related large-subunit catalase KatE from *E. coli* showed that mutations in the elongated main channel have a reductive effect on catalytic turnover of no more than 50% (Jha *et al.*, 2012 ▸).

**Figure 2:**
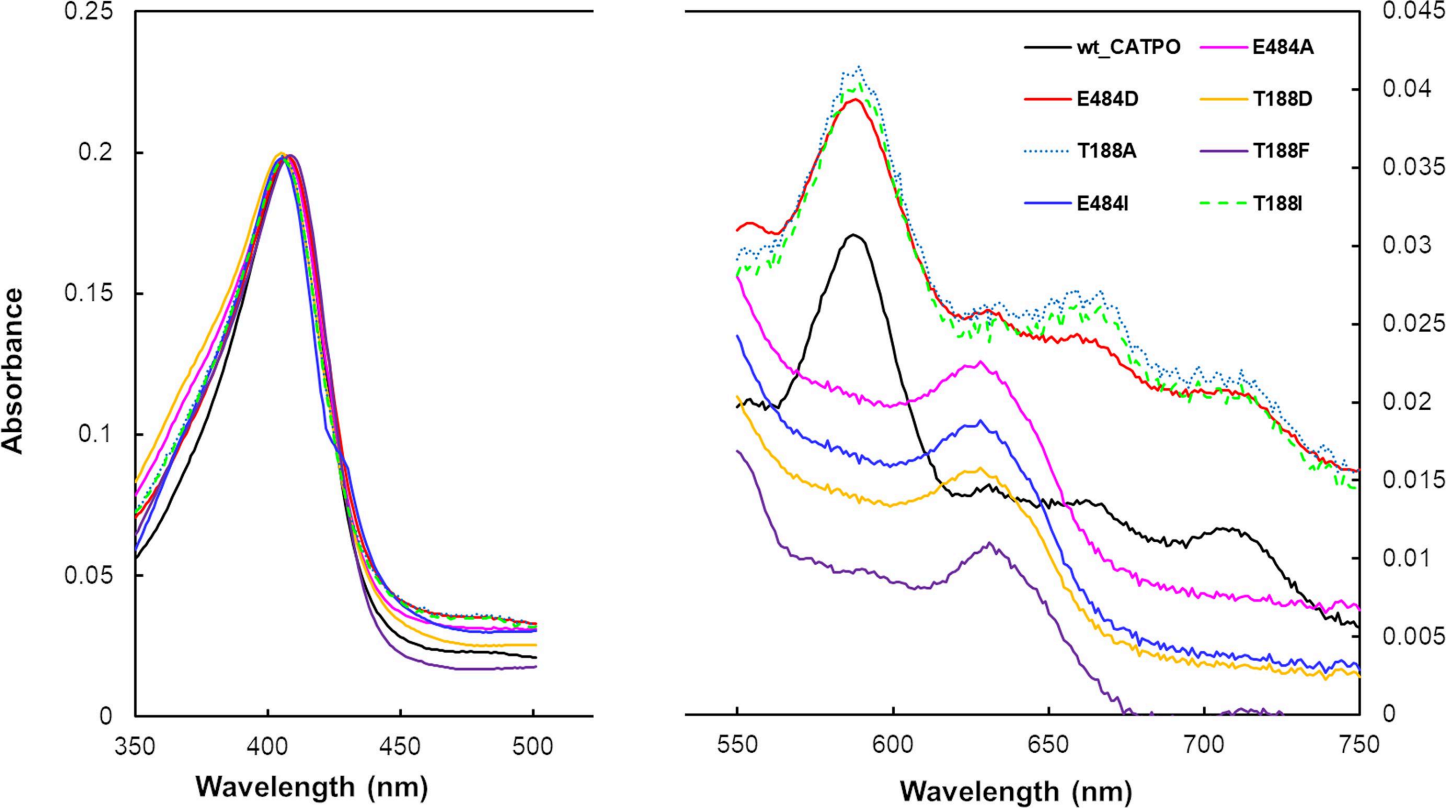
Absorbance spectra of CATPO and its E484A, E484I, E484D, T188A, T188D, T188F and T188I variants in 0.1 *M* sodium phosphate buffer pH 7.0 at room temperature. The wild-type CATPO spectrum is fitted so that the Soret peak for each of the variants has an equivalent value. Full spectra are provided in Supplementary Fig. S10.

In addition to kinetic analysis, UV–Vis spectra were recorded. Fig. 2 ▸ shows the spectra of CATPO and the variants of interest. Catalase enzymes are characterized by strong absorption in the Soret band, with an Rz (Reinheitszahl) value (the *A*_407_/*A*_280_ ratio) of close to 1 (Zámocký & Koller, 1999 ▸). As can be seen in Fig. 2 ▸, all variants displayed the expected peak in the Soret region around *A*_407_. However, differences in Rz values were observed for some variants, including E484I, T188D and T188I (Table 3 ▸). Lower Rz values are likely to be explained by nonstoichiometric heme binding, as all enzymes were purified to at least 95% purity. Previous studies have reported iron-deficient catalase folds, including cases where the porphyrin ring is present but the iron itself is absent. For example, it has been shown that the prosthetic group of the recombinant *Proteus mirabilis* catalase (PMC) lacks heme but contains protoporphyrin IX. The content of protoporphyrin IX depended on the expression conditions, and the absence of heme had no effect on the specific heme reactivity. In PMC catalase this is explained by the replacement of the heme group by protoporphyrin IX (Andreoletti *et al.*, 2003 ▸).

Catalases contain either heme *b* or heme *d* (an oxidized form of heme *b*) in their active centre. As can be seen in Fig. 2 ▸, the recombinant wild-type CATPO enzyme and its E484D and T188A variants have absorption peaks at 590 and 715 nm (Loewen *et al.*, 1993 ▸), indicating that these enzymes contain a *d*-type heme as a prosthetic group. Mutation of the Glu484 and Thr188 residues to Ala484, Ile484, Asp188, Phe188 or Ile188, on the other hand, resulted in a new absorption maximum at 630 nm, indicating that these variants contain heme *b* (protoheme). The presence of heme *d* in the T188A variant and of heme *b* in the E484A and T188F variants was also confirmed by crystallographic analysis (Supplementary Fig. S2).

In fact, heme is synthesized as protoheme (heme *b*), but in some large-subunit catalases this is converted to heme *d* by the enzyme itself using H_2_O_2_ as an oxidant (Timkovich & Bondoc, 1990 ▸; Loewen *et al.*, 1993 ▸). It has been shown that heme oxidation is not necessary for catalytic activity, but it could play a role in making compound I (a high-valent iron intermediate) more stable (Díaz *et al.*, 2012 ▸). Consistent with this, CATPO variants containing heme *d* had similar *k*_cat_ values to the wild-type enzyme (see Table 3 ▸).

The E484A, E484I and T188I variants were tested to see whether heme *b* could be oxidized to heme *d* using ascorbate, as previously shown for KatE (Loewen *et al.*, 1993 ▸). However, no conversion was observed (data not shown). This suggests that the nature of the residues at positions 484 and 188 is important for heme oxidation. Similar results have also been reported for the HPII enzyme (Jha *et al.*, 2012 ▸).

### 3.2. Characterization of the oxidase activities of the Glu484 and Thr188 variants

We have previously shown that CATPO has an oxidase activity that acts on phenolic compounds with two hydroxyl groups, especially in *ortho* positions. This activity is oxygen-dependent but is independent of hydrogen peroxide. The highest activity is shown towards catechol (Sutay Kocabas *et al.*, 2008 ▸; Koclar Avci *et al.*, 2013 ▸; Yuzugullu, Trinh, Smith *et al.*, 2013 ▸). Oxidase activity has also been documented in purified catalases extracted from mammalian cells (Vetrano *et al.*, 2005 ▸), *Bacillus pumilus* (Sangar *et al.*, 2012 ▸), *Thermobifida fusca* (Lončar & Fraaije, 2015 ▸) and *Amaranthus cruentus* (Teng *et al.*, 2016 ▸; Chen *et al.*, 2017 ▸), and in various commercially available catalase samples from human erythrocytes, bovine liver and *Corynebacterium glutamicum*, as well as from another fungal source, *Aspergillus niger* (Yuzugullu, Trinh, Smith *et al.*, 2013 ▸).

The phenol oxidase activity of the Glu484 and Thr188 variants was investigated. The phenol oxidase activity was not affected by the addition of hydrogen peroxide (1–100 m*M*) or ethanol (2–200 m*M*) either before or after the addition of catechol to the reaction medium, indicating that the reaction is oxidative and not peroxidative (Supplementary Fig. S3). Catechol oxidation is saturable (Supplementary Fig. S4) and reversible. The *K*_m_ and *k*_cat_ values for the oxidation of catechol by wild-type CATPO are 33 ± 0.5 m*M* and 7.3 ± 0.4 × 10^3^ s^−1^, respectively. The *K*_m_ of the variants showed minor changes compared with the wild-type enzyme. However, the turnover numbers increased for the E484A (sixfold), E484I (fourfold), T188D (threefold), T188F (fourfold) and T188I (fourfold) variants, while they did not change significantly for the E484D and T188A variants. As a result, the catalytic efficiencies of the E484A, E484I, T188D, T188F and T188I variants were significantly higher than those of wild-type CATPO and the E484D and T188A variants (Table 3 ▸; Supplementary Fig. S5).

Our results show that the turnover numbers and catalytic efficiencies were significantly altered in some CATPO variants. The CATPO variants with increased phenol oxidase turnover all contain heme *b* (with two carboxyl chains in its structure) rather than heme *d* (with one carboxyl chain and one *cis*-hydroxyspirolactone in its structure). In our previous report, it was also shown that phenol oxidase activity is increased in heme-*b*-containing lateral channel variants (Goc *et al.*, 2021 ▸).

Additional experiments were performed with the CATPO variants using different phenolic substrates to determine whether there was any change in substrate specificity. Since catechol is a common polyphenol oxidase (PPO) substrate (Panadare & Rathod, 2018 ▸), other PPO substrates such as 4-methylcatechol, 1,4-*tert*-butylcatechol, pyrogallol, *p*-hydroquinone and ABTS (Supplementary Fig. S6) were also tested.

As shown in Table 4 ▸, all variants showed phenol oxidase activity for all substrates tested. The *K*_m_ values for all substrates tested were similar to that of wild-type CATPO. However, the *V*_max_ and thus the catalytic efficiency (*k*_cat_/*K*_m_) of the E484A, E484I, T188D, T188F and T188I variants was markedly higher than those of the wild-type enzyme for catechol, 4-methylcatechol, 1,4-*tert*-butylcatechol and pyrogallol. When comparing the activity of CATPO and its variants towards the tested substrates, all exhibited a similar profile towards the substrates. Accordingly, the substrate selectivity for all variants is as follows: catechol > 4-methylcatechol > 1,4-*tert*-butylcatechol > pyrogallol > hydroquinone > ABTS.

The ability of the alternate polyphenol oxidase substrates to compete with catechol for the oxidase activity of CATPO and its variants was also tested. All of the alternate polyphenolic substrates inhibited the oxidation of catechol in a competitive manner (Supplementary Fig. S7). The most potent inhibitor among them was 4-methylcatechol (*K*_i_ = 1.5 ± 0.4 m*M* for wild-type CATPO), followed by 1,4-*tert*-butylcatechol (*K*_i_ = 2.1 ± 0.8 m*M*), pyrogallol (*K*_i_ = 4.2 ± 0.8 m*M*), *p*-hydroquinone (*K*_i_ = 8.5 ± 0.5 m*M*) and ABTS (*K*_i_ = 11 ± 1.0 m*M*) (Table 5 ▸). This is consistent with previously reported substrate-specificity analyses, which indicated that the CATPO enzyme predominantly acts on *ortho*-diphenols (Table 4 ▸; Sutay Kocabas *et al.*, 2008 ▸). In the CATPO variants, the same phenolic compounds also competitively inhibited catechol oxidation (*i.e.* ABTS; Table 5 ▸, Supplementary Fig. S8). The *K*_i_ values calculated were similar for the E484D and E484I variants. On the other hand, the catechol oxidase activity of the E484A, E484I, T188D, T188F and T188I variants showed a much lower sensitivity to inhibition by the other polyphenolic substrates. Similar to CATPO, the inhibitory effect of phenols is as follows: 4-methylcatechol > 1,4-*tert*-butylcatechol > pyrogallol > *p*-hydroquinone > ABTS.

Several catalase and polyphenol oxidase inhibitors were also examined. Halides have been reported to inhibit catalase and oxidases (Thibodeau & Keefe, 1990 ▸; Xu, 1996 ▸). For wild-type CATPO, we found that sodium fluoride was an uncompetitive inhibitor (Supplementary Fig. S7), with a *K*_i_ of 0.67 ± 0.1 m*M* (Table 5 ▸). In a similar study with mammalian catalase, the *K*_i_ value for NaF was reported to be 0.75 m*M* and it was described as an uncompetitive inhibitor (Vetrano *et al.*, 2005 ▸). Salicylhydroxamic acid, a polyphenol oxidase inhibitor, is also a competitive inhibitor of the catechol oxidase activity of CATPO (*K*_i_ = 2.2 ± 0.4 m*M*). On the other hand, the typical catalase inhibitors 3-amino-1,2,4-triazole (3TR) and sodium azide are weaker competitive inhibitors of the catechol oxidase activity of CATPO, with *K*_i_ values of 21 ± 1.0 m*M* and 42 ± 1.2 m*M*, respectively.

When we examined the effects of inhibitors on the oxidase activity of CATPO variants, the mutations did not result in a change in the type of inhibition (*i.e.* NaF; Supplementary Fig. S9). Sodium azide and 3TR did not have as strong an inhibitory effect as on CATPO. Salicylichydroxamic acid caused inhibition in all variants, with *K*_i_ values between 2 and 6.7 m*M*. NaF also caused inhibition in all variants, but its effect was stronger on the E484D (*K*_i_ = 0.66 m*M*) and T188A (*K*_i_ = 0.65 m*M*) variants.

### 3.3. Structural characterization of Glu484 and Thr188 variants

To better understand whether the changes observed in the catalytic activities of the variants are due to an altered solvent occupancy of the channels (especially the main channel) that extend to the active site, the crystal structures of the three variants E484A, T188F and T188A were determined. The E484A and T188F variants were selected because they have a lower catalase *k*_cat_ but a higher phenol oxidase activity than CATPO. The T188A variant was selected because it has a similar catalase *k*_cat_ value to CATPO.

**Figure 3:**
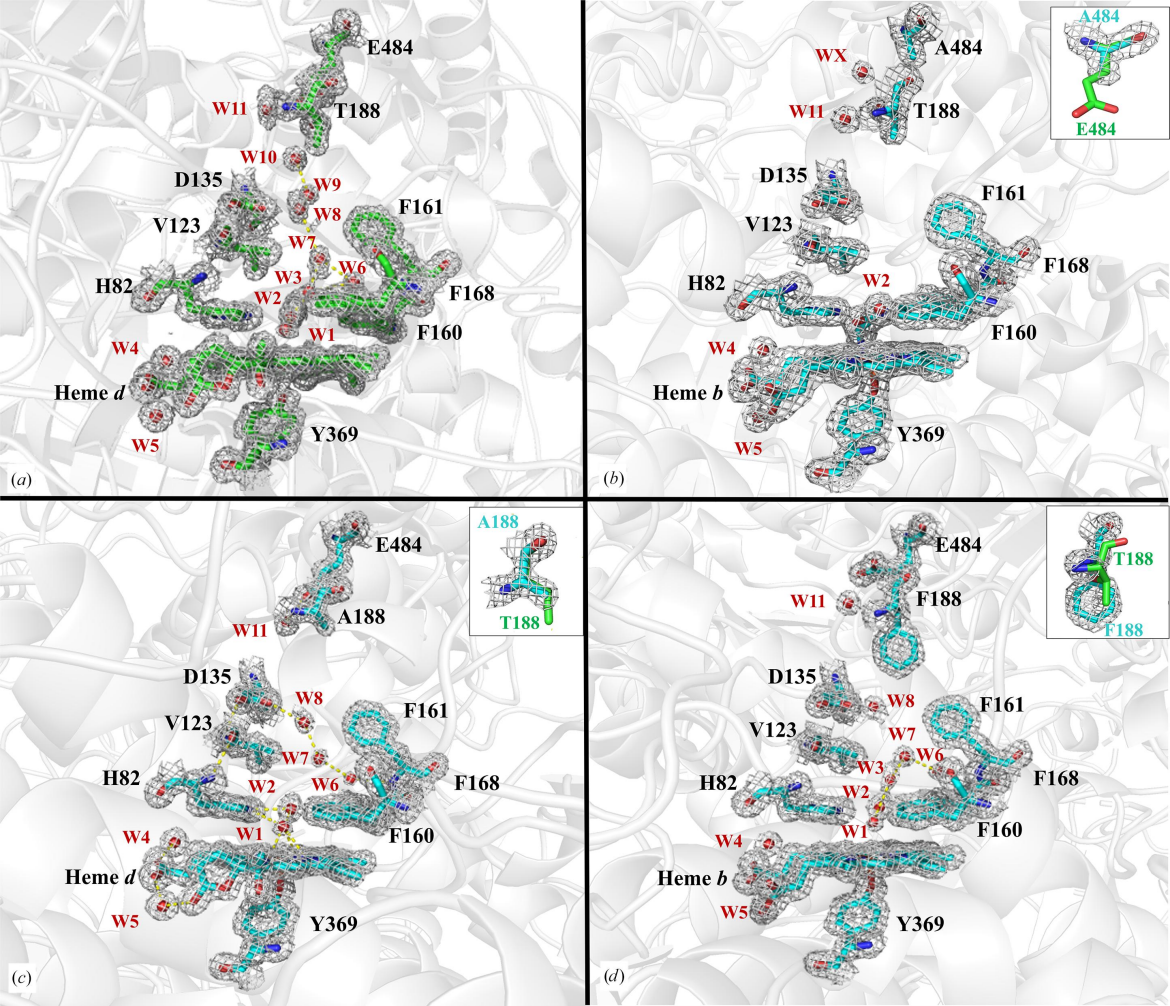
Comparison of the main channel and heme cavity of wild-type CATPO (*a*) and its E484A (*b*), T188A (*c*) and T188F (*d*) variants. The corresponding omit maps for residues, ligands and waters are shown for the four cases at 1.0 r.m.s.d. The gate residues Glu484 and Thr188 are located at the top of each figure at the entry to the main channel. Also shown are the conserved residues lining the channel and the heme cavity: His82, Val123, Asp135, Phe160, Phe161, Phe168 and Tyr369. The ring of hydrophobic residues that includes Val123 defines the narrowest point of the main channel. Heme *d* is found in wild-type CATPO (*a*) and the T188A variant (*c*), whereas heme *b* is found in the E484A (*b*) and T188F (*d*) variants. Changes in solvent organization are evident in the structures. The water molecules of the recombinant wild-type enzyme and their equivalents in the variants are labelled numerically: W1–W11. The water molecule WX in the E484A variant does not correspond to any water in recombinant wild-type CATPO. The insets show the difference in the side-chain geometry of the mutated and nonmutated residues.

The mutated side chains at positions 484 and 188 were evident for the E484A, T188A and T188F variants (Fig. 3 ▸). When comparing the crystal structures of CATPO and its three variants, some differences can be noted. The first is the absence of the polar contact between Glu484 and Thr188 in the three variants (Fig. 3 ▸). However, the water molecule (W11) to which Thr188 forms a hydrogen bond was retained in all variants.

The second difference was observed in the solvent chain in the main channel (Fig. 3 ▸). Of the three variants, T188A has the highest similarity to CATPO in terms of solvent-chain integrity in the main channel. The fact that their catalytic activities are similar and that both contain *d*-type heme groups is consistent with this structural similarity. The most extreme example is the E484A variant, which lacks six water molecules in the main channel (Fig. 3 ▸). Although the *K*_m_ value did not change compared with the wild-type CATPO enzyme, this mutation had a negative effect on the catalytic activity. In the T188F mutant the solvent distribution in the upper part of the main channel is very different as the phenylalanine protrudes into the channel, while the lower part of the channel is similar to that of wild-type CATPO (Fig. 3 ▸). From the results of the structural comparison, it can be concluded that there is no correlation between the diameter of the channel and the water occupancy, as all three mutations disrupt the ordered solvent distribution. This hypothesis is supported by the study reported by Jha *et al.* (2012 ▸), in which it was shown that although the S234A and E530I variants did not show significant changes in the main-channel diameter, nearly all water molecules were in the channel in the S234A variant, whereas almost no solvent was observed in the E530I variant.

It was also found that there are slight differences in the solvent structures of the four subunits in the E484A, T188A and T188F variants (Supplementary Table S3). When the *B* factors of the 11 water molecules in the main channel of wild-type CATPO (PDB entry 4aum) were analysed, it was found that the water molecules were well ordered in the structure of each subunit. Comparison of the superimposed structures of the T188A and T188F mutants with wild-type CATPO showed that W1 had a high *B* factor. In addition, neither mutant had similar water molecules to wild-type CATPO. However, when the E484A mutant was superimposed with the wild-type enzyme and the water molecules were compared, it was found that a significant number of water molecules were missing. Although this could indicate that the data from the crystal of the E848A mutant were of insufficiently high resolution, examination of the *B* factors showed that the water molecules were well ordered, indicating a well elaborated structure.

**Figure 4:**
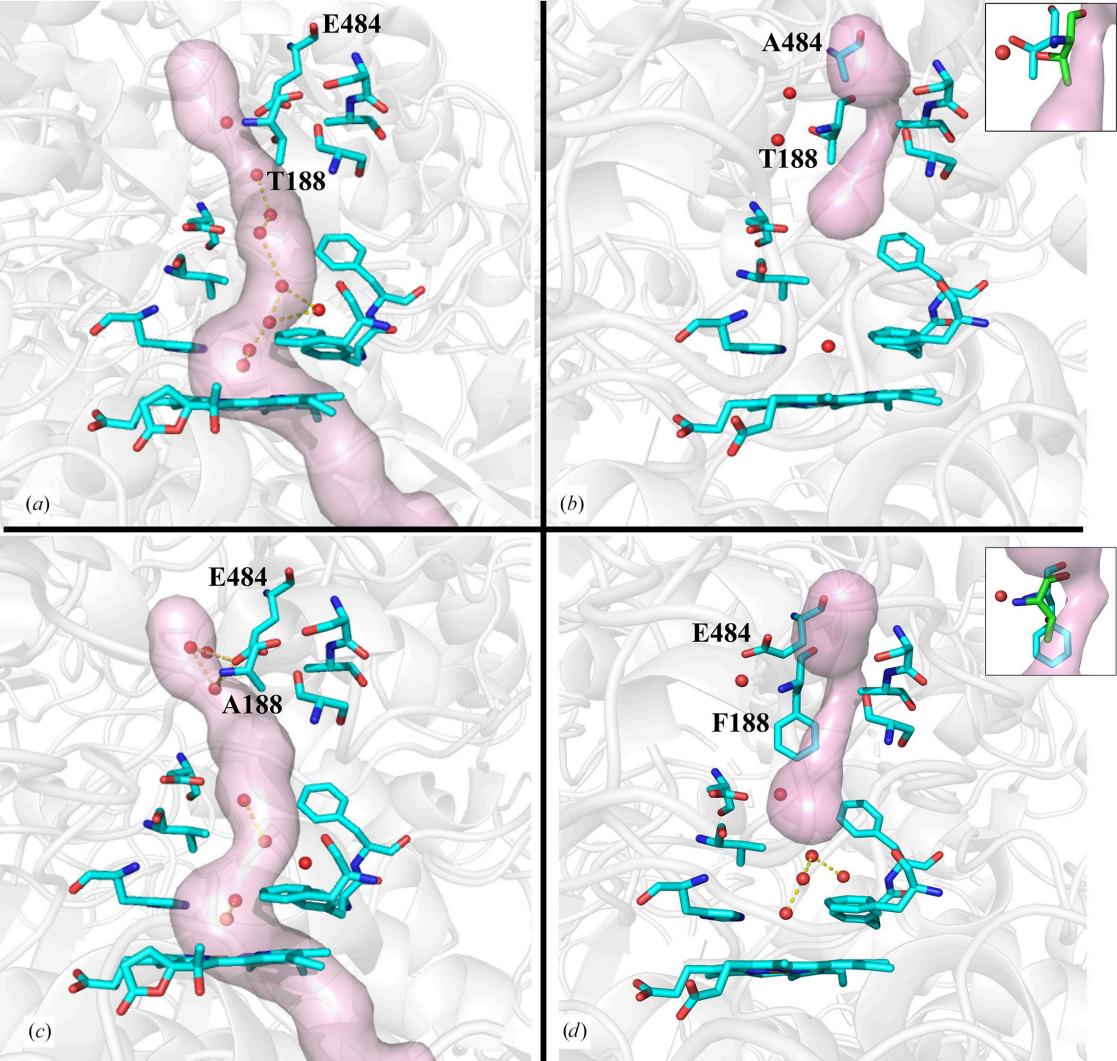
Main-channel architecture in recombinant wild-type CATPO (*a*) and its E484A (*b*), T188A (*c*) and T188F (*d*) variants in subunit *A* created using *Caver*. The channel in the T188A mutant is similar to that of wild-type CATPO, while in the E484A and T188F mutants the channel is disrupted and is likely to be effectively closed. The same is seen in all subunits. The insets show the shift in the position of Thr188 (cyan) and Phe188 (cyan) in the E484A (*b*) and T188F (*d*) variants compared with the corresponding position (Thr188, pink) in wild-type CATPO, respectively.

The other structural difference observed was that the E484A and T188F variants appear to form a second, smaller cavity at the upper right of the main channel in comparison to the wild-type enzyme (Fig. 4 ▸). The approximately 40% decrease in the catalase activity of the E484A and T188F variants compared with CATPO could be due to the formation of smaller cavities in the main channel after the mutations.

The channel architecture also differs in CATPO and its three variants, as shown in Fig. 4 ▸. The channel appears to be closed in the E484A and T188F variants, which would prevent H_2_O_2_ molecules from entering the cavity. This is due to a shift in the position of Thr188 (in the E484A variant) into the channel and the corresponding Phe188 residue in the T188F variant protruding deeply into the channel (Fig. 4 ▸). Considering that relatively lower *k*_cat_ but similar *K*_m_ values were measured in these variants compared with the wild-type enzyme (Table 3 ▸), we suspect that H_2_O_2_ is present in the main channel for catalytic activity but insufficient H_2_O_2_ is retained in the heme cavity for efficient heme oxidation. In all monofunctional catalases heme *b* is synthesized first and is then oxidized to heme *d* with the help of H_2_O_2_ in large-subunit catalases (Loewen *et al.*, 1993 ▸). This indicates that H_2_O_2_ plays an important role in heme oxidation. In addition, previous reports have shown that H_2_O_2_ must be retained in the active pocket for the second step of the catalytic reaction (Domínguez *et al.*, 2010 ▸; Jha *et al.*, 2012 ▸; Sevinc *et al.*, 1999 ▸). Related to this, the conversion of Val228 in CATPO and Ile274 in HPII to smaller amino acids resulted in lower activity, consistent with the retention of H_2_O_2_ in the heme cavity (Goc *et al.*, 2021 ▸; Jha *et al.*, 2012 ▸).

The presence of ordered solvent chains was observed in the lower part of the main channel and in the cavity in the T188F variant, but not in the E484A variant (Figs. 3 ▸ and 4 ▸). Despite differences in solvent-chain structures, both variants show a similar phenol oxidase activity (Table 3 ▸). We have previously shown that there is a binding pocket for phenol oxidase substrates in the lateral channel, where the nicotinamide moiety of NADPH is present in NADPH-binding catalases. The amino-acid changes in this channel showed that the catalase and phenol oxidase activities can be differentially affected. Mutations that open the channel entrance led to a decrease in catalase activity, indicating that the storage of hydrogen peroxide as an electron acceptor in the active site was restricted. On the other hand, an increase in phenol oxidase activity was observed independent of catalase activity, indicating that the mutation brought the appropriate electron donor closer to the heme pocket (Yuzugullu Karakus *et al.*, 2018 ▸; Goc *et al.*, 2021 ▸). Reports that phenol oxidase activity is independent of H_2_O_2_ (Ögel *et al.*, 2006 ▸; Yuzugullu, Trinh, Smith *et al.*, 2013 ▸) and that phenol oxidative substrates prefer the lateral channel rather than the main channel support this hypothesis (Yuzugullu Karakus *et al.*, 2018 ▸).

## 4. Conclusions

Here, we investigated the role of the gate residues Glu484 and Thr188 in the catalase enzyme from *S. thermophilum* using spectroscopic, kinetic and structural data on different variants generated by site-directed mutagenesis. The most interesting result was that the E484A, E484I, T188D, T188F and T188I variants were still catalytically active and possessed higher phenol oxidase activities than wild-type CATPO, although they were associated with heme *b* rather than heme *d* as in the wild-type enzyme. An increase in phenol oxidase activity in some heme *b*-containing variants upon the mutation of catalytically nonessential residues has previously been reported (Goc *et al.*, 2021 ▸). Further kinetic analysis of the phenol oxidase activity also confirmed previous reports that this catalase enzyme can utilize a wide range of oxidase substrates (Ögel *et al.*, 2006 ▸; Sutay Kocabas *et al.*, 2008 ▸). Additionally, the kinetic results supported our previous observations that the phenol oxidase activity is independent of the presence of H_2_O_2_ (Yuzugullu Karakus *et al.*, 2018 ▸; Goc *et al.*, 2021 ▸).

Another conclusion is that the five heme *b*-containing variants (although two of them, E484I and T188I, appear to have a very low heme content) generally have a lower sensitivity to the inhibitory effect of phenolics than CATPO. This supports our previous observation (Yuzugullu Karakus *et al.*, 2018 ▸) that the inhibitory effect of the catalase inhibitor 3TR was reduced on increasing the catechol concentration. This suggests that the phenol oxidase activity might have a similar protective effect on the enzyme as NADPH for mammalian catalases (Vetrano *et al.*, 2005 ▸).

Although the T188A mutation did not result in a significant change in the catalytic activity, overall it can be concluded that despite their distance from the active site, Glu484 and Thr188 have an indirect effect on the catalase enzyme by influencing both the secondary phenol oxidase activity and the nature of the heme cofactor.

## Supplementary Material

PDB reference: *Scytalidium thermophilum* catalase, T188F mutant, 5yem

PDB reference: E484A mutant, 7wca

PDB reference: T188A mutant, 7vn0

Supplementary Tables and Figures. DOI: 10.1107/S2059798323011063/gm5099sup1.pdf

## Figures and Tables

**Table 1:** Oligonucleotides used to mutate the *catpo* gene in the pET-28-TEV-CATPO plasmid

Variant	Sequence change	Oligonucleotide†
E484A	GAA→GCA	5′-CCTGGTTAACGCTATGCGTTTC**GCA**ATCTCTCTGGTTAAATCTG
E484D	GAA→GAC	5′-CCTGGTTAACGCTATGCGTTTC**GAC**ATCTCTCTGGTTAAATCTG
E484I	GAA→ATC	5′-CCTGGTTAACGCTATGCGTTTC**ATC**ATCTCTCTGGTTAAATCTG
T188A	ACC→GCC	5′-CCCGCAGGCTGCT**GCC**GCTCACGACTCTGCTTGGG
T188D	ACC→GAC	5′-CCCGCAGGCTGCT**GAC**GCTCACGACTCTGCTTGGG
T188F	ACC→TTC	5′-CCCGCAGGCTGCT**TTC**GCTCACGACTCTGCTTGGG
T188I	ACC→ATC	5′-CCCGCAGGCTGCT**ATC**GCTCACGACTCTGCTTGGG

† The mutated sequence is shown in bold.

**Table 2:** Crystallographic data-collection and refinement statistics for the main-channel variants

Variant	E484A	T188A	T188F
PDB code	7wca	7vn0	5yem
Data collection			
Beamline at ESRF	ID29	ID29	ID29
Wavelength (Å)	0.97	0.97	0.97
Temperature (K)	100	100	100
Space group	*I*2	*I*2	*I*2
*a*, *b*, *c* (Å)	124.74, 120.20, 84.08	124.94, 120.79, 184.75	125.11, 121.54, 186.02
Resolution (Å)	46.38–1.78 (1.81–1.78)	112.6–1.40 (1.42–1.40)	112.9–1.49 (1.51–1.49)
*R*_merge_ † (%)	8.4 (75.6)	7.0 (70.8)	9.2 (81.2)
*R*_p.i.m._ ‡ (%)	8.2 (74.0)	6.4 (64.0)	8.4 (72.0)
CC_1/2_	0.995 (0.736)	0.997 (0.548)	0.996 (0.555)
Observed reflections	752331 (37914)	1774576 (72380)	1503729 (68451)
Unique reflections	250722 (12394)	520328 (23479)	440118 (20656)
Completeness (%)	98.9 (98.9)	98.9 (93.1)	99.3 (94.4)
Multiplicity	3.0 (3.1)	3.4 (3.1)	3.4 (3.3)
〈*I*/σ(*I*)〉	8.0 (1.9)	9.2 (1.3)	7.4 (1.4)
Refinement			
*R* factor (%)	15.8	15.8	15.8
*R*_free_ § (%)	19.7	18.2	18.7
No. of protein atoms	21231	21968	22552
No. of solvent molecules	1678	1983	2386
No. of ligand atoms	187	181	182
Average overall *B* factor (Å^2^)	26.5	14.06	13.48
R.m.s.d., bond lengths¶ (Å)	0.019	0.023	0.021
R.m.s.d., bond angles¶ (°)	1.863	2.199	1.968
Ramachandran analysis†† (%)			
Residues in most favoured regions	97.07	97.18	96.78
Outliers	0.19	0.0	0.19
Alignment with wild-type structure‡‡ (PDB entry 4aum; Yuzugullu, Trinh, Smith *et al.*, 2013 ▸) over all residues			
R.m.s.d. (Å)	0.251	0.262	0.32
*Q*-score	0.981	0.979	0.976

† *R*_merge_ = 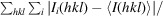 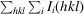 .

‡ *R*_p.i.m._ is the precision-indicating (multiplicity-weighted) *R*_merge_.

§ *R*_free_ was calculated with 5% of the reflections that were set aside randomly.

¶ Based on the ideal geometry values of Engh & Huber (1991 ▸).

†† Ramachandran analysis using *MolProbity* (Chen *et al.*, 2010 ▸).

‡‡ The r.m.s.d and *Q*-score were calculated using *GESAMT* (Krissinel, 2012 ▸).

**Table 3:** Kinetic comparison of the CATPO variants Rz = *A*_407_/*A*_280_ indicates the heme content of the enzyme. 5 µ*M* catalase enzyme was used for kinetic assays. Normalized kinetic constants according to the heme content of each variant are given. Detailed normalized and unnormalized data are provided in Supplementary Table S2. Maximum absorption peaks at 590 nm (heme *d*) and 630 nm (heme *b*) were examined to determine the heme type; heme *d*-containing variants are shown in bold for ease of viewing. Hydrogen peroxide and catechol were used as substrates for the catalase and phenol oxidase activities, respectively.

	Catalase activity†			Phenol oxidase activity†				
	*K*_m,app_ ‡ (m*M*)	*k*_cat_ (s^−1^)	*k*_cat_/*K*_m,app_ ‡ (s^−1^ *M* ^−1^) (×10^2^)	*K*_m_ (m*M*)	*k*_cat_ (s^−1^)	*k*_cat_/*K*_m_ (s^−1^ *M* ^−1^) (×10^2^)	Rz	Heme
**CATPO**	**10 ± 2.0**	**203000 ± 6300**	**203000 ± 4900**	**33 ± 0.5**	**7300 ± 365**	**2200 ± 130**	**0.8**	***d***
E484A	8 ± 0 5	95000 ± 2000	119000 ± 908	30 ± 1.0	43500 ± 2600	14500 ± 130	0.8	*b*
**E484D**	**11 ± 1.0**	**192000 ± 4600**	**175000 ± 1600**	**35 ± 0.5**	**7400 ± 296**	**2100 ± 105**	**0.8**	***d***
E484I	18 ± 2.0	216000 ± 3800	120000 ± 1580	30 ± 1.0	26500 ± 1325	8800 ± 72	0.5	*b*
**T188A**	**11 ± 0.5**	**209000 ± 3100**	**190000 ± 1050**	**33 ± 0.5**	**6700 ± 335**	**2000 ± 120**	**0.7**	***d***
T188D	17 ± 1.0	212000 ± 5300	125000 ± 924	25 ± 1.5	22300 ± 669	8900 ± 70	0.5	*b*
T188F	6 ± 2.0	105000 ± 1470	175000 ± 5065	30 ± 1.0	30600 ± 1800	10200 ± 220	0.8	*b*
T188I	21 ± 1.0	242000 ± 2400	115000 ± 650	31 ± 0.5	28400 ± 1100	9200 ± 45	0.4	*b*

† One unit of catalase is defined as the amount that catalyses the degradation of 1 µmol H_2_O_2_ in 1 min. One unit of phenol oxidase is defined as the amount that catalyses the formation of 1 nmol product per minute.

‡ *K*_m,app_ is the H_2_O_2_ concentration at *V*_max_/2 and is used because the catalase reaction does not saturate with substrate and therefore does not precisely follow Michaelis–Menten kinetics (Switala & Loewen, 2002 ▸).

**Table:** 

	CATPO			E484A		
Substrate	*K*_m_ (m*M*)	*V*_max_ (m*M* min^−1^)	*k*_cat_/*K*_m_ (s^−1^ *M* ^−1^) (×10^2^)	*K*_m_ (m*M*)	*V*_max_ (m*M* min^−1^)	*k*_cat_/*K*_m_ (s^−1^ *M* ^−1^) (×10^2^)
Catechol	33 ± 0.5	25000 ± 763	2200 ± 130	30 ± 1.0	88000 ± 880	14500 ± 130
4-Methylcatechol	35 ± 0.3	21320 ± 845	1580 ± 110	33 ± 1.0	75500 ± 550	10100 ± 90
1,4-*tert*-Butylcatechol	36 ± 1.0	20000 ± 362	1240 ± 90	33 ± 0.5	65000 ± 745	10000 ± 65
Pyrogallol	40 ± 1.8	18450 ± 451	960 ± 65	36 ± 0.7	50000 ± 156	6000 ± 50
Hydroquinone	45 ± 1.5	10000 ± 300	750 ± 50	43 ± 1.2	17800 ± 100	600 ± 40
ABTS	50 ± 1.5	5620 ± 250	590 ± 20	46 ± 1.0	8600 ± 220	300 ± 10

**Table:** 

	E484D			E484I		
Substrate	*K*_m_ (m*M*)	*V*_max_ (m*M* min^−1^)	*k*_cat_/*K*_m_ (s^−1^ *M* ^−1^) (×10^2^)	*K*_m_ (m*M*)	*V*_max_ (m*M* min^−1^)	*k*_cat_/*K*_m_ (s^−1^ *M* ^−1^) (×10^2^)
Catechol	35 ± 0.5	23000 ± 1500	2100 ± 105	30 ± 1.0	50000 ± 1988	8800 ± 72
4-Methylcatechol	36 ± 0.1	20600 ± 712	1500 ± 88	35 ± 0.7	41500 ± 840	5480 ± 80
1,4-*tert*-Butylcatechol	38 ± 0.5	18800 ± 144	1300 ± 100	37 ± 0.4	28600 ± 430	4450 ± 66
Pyrogallol	40 ± 0.5	16800 ± 300	600 ± 50	40 ± 1.1	20300 ± 330	3060 ± 30
Hydroquinone	46 ± 0.8	9900 ± 125	300 ± 50	45 ± 1.3	10000 ± 100	460 ± 20
ABTS	53 ± 1.0	5500 ± 132	100 ± 10	50 ± 0.5	4600 ± 98	170 ± 10

**Table:** 

	T188A			T188D		
Substrate	*K*_m_ (m*M*)	*V*_max_ (m*M* min^−1^)	*k*_cat_/*K*_m_ (s^−1^ *M* ^−1^) (×10^2^)	*K*_m_ (m*M*)	*V*_max_ (m*M* min^−1^)	*k*_cat_/*K*_m_ (s^−1^ *M* ^−1^) (×10^2^)
Catechol	33 ± 0.5	22500 ± 1000	2000 ± 120	25 ± 1.5	40000 ± 4000	8900 ± 70
4-Methylcatechol	35 ± 1.0	21000 ± 880	1640 ± 210	30 ± 0.1	28000 ± 970	7620 ± 100
1,4-*tert*-Butylcatechol	37 ± 1.0	19400 ± 568	1460 ± 150	38 ± 0.5	18000 ± 500	6590 ± 150
Pyrogallol	40 ± 1.0	16200 ± 410	660 ± 88	41 ± 1.0	15000 ± 150	5710 ± 200
Hydroquinone	46 ± 0.4	9850 ± 85	300 ± 30	46 ± 1.0	9600 ± 65	480 ± 64
ABTS	52 ± 0.8	5000 ± 100	90 ± 5	55 ± 1.0	4000 ± 88	200 ± 25

**Table:** 

	T188F			T188I		
Substrate	*K*_m_ (m*M*)	*V*_max_ (m*M* min^−1^)	*k*_cat_/*K*_m_ (s^−1^ *M* ^−1^) (×10^2^)	*K*_m_ (m*M*)	*V*_max_ (m*M* min^−1^)	*k*_cat_/*K*_m_ (s^−1^ *M* ^−1^) (×10^2^)
Catechol	30 ± 1.0	70000 ± 1290	10200 ± 220	31 ± 0.5	65000 ± 1100	9200 ± 45
4-Methylcatechol	32 ± 1.0	54150 ± 800	6570 ± 560	34 ± 1.2	43000 ± 900	6600 ± 600
1,4-*tert*-Butylcatechol	34 ± 0.4	41600 ± 340	5230 ± 145	35 ± 0.5	30500 ± 650	4900 ± 410
Pyrogallol	37 ± 0.5	28100 ± 760	3930 ± 250	38 ± 0.5	27400 ± 310	3470 ± 256
Hydroquinone	42 ± 1.0	13000 ± 140	530 ± 65	44 ± 0.4	11000 ± 110	490 ± 78
ABTS	45 ± 0.5	6600 ± 120	270 ± 25	48 ± 0.7	6000 ± 100	190 ± 18

**Table 5:** Comparison of the effects of various phenolics on catechol oxidation by CATPO and its variants Polyphenolic alternate substrates are shown in bold.

	*K*_i_ (m*M*)							
Inhibitor	CATPO	E484A	E484D	E484I	T188A	T188D	T188F	T188I
Competitive								
** Pyrogallol**	**4.2 ± 0.8**	**71 ± 4.0**	**4.1 ± 0.7**	**58 ± 1.8**	**4.1 ± 0.8**	**55 ± 5.5**	**64 ± 6.0**	**62 ± 2.5**
** 4-Methylcatechol**	**1.5 ± 0.4**	**55 ± 5.0**	**1.5 ± 0.1**	**46 ± 2.0**	**1.5 ± 0.4**	**50 ± 2.5**	**45 ± 4.8**	**40 ± 2.4**
** 1,4-*tert*-Butylcatechol**	**2.1 ± 0.8**	**67 ± 7.0**	**2.2 ± 0.5**	**50 ± 1.5**	**2.1 ± 0.1**	**49 ± 3.2**	**59 ± 4.5**	**53 ± 4.5**
** ABTS**	**11 ± 1.0**	**96 ± 5.4**	**12 ± 1.2**	**81 ± 8.0**	**12 ± 1.2**	**81 ± 1.0**	**84 ± 7.0**	**82 ± 2.5**
** Hydroquinone**	**8.5 ± 0.5**	**81 ± 4.7**	**8.5 ± 1.4**	**70 ± 3.5**	**8.6 ± 0.6**	**66 ± 5.5**	**76 ± 5.1**	**70 ± 4.0**
Salicylhydroxamic acid	2.2 ± 0.4	6.7 ± 1.1	2.0 ± 0.4	5.4 ± 0.5	2.2 ± 0.4	5.4 ± 0.4	5.5 ± 0.7	5.4 ± 0.9
Sodium azide	42 ± 1.2	74 ± 4.8	41 ± 4.1	56 ± 1.6	42 ± 1.4	55 ± 5.0	62 ± 2.5	60 ± 6.0
3TR	21 ± 1.0	65 ± 5.5	22 ± 3.2	40 ± 4.0	20 ± 0.2	41 ± 4.1	54 ± 4.5	42 ± 4.0
Uncompetitive								
NaF	0.67 ± 0.1	7.8 ± 1.1	0.66 ± 0.1	5.5 ± 0.2	0.65 ± 0.1	5.0 ± 0.5	6.4 ± 0.4	5.8 ± 0.4
