# Supplementary material for: Investigation of how gate residues in the main channel affect the catalytic activity of Scytalidium thermophilum catalase
Source: Acta Crystallogr D Struct Biol. 2024 Jan 24;80(Pt 2):101–12. doi: 10.1107/S2059798323011063 (PMC10836395; doi:10.1107/S2059798323011063)
Supplement: Supplementary file 1 [file d-80-00101-sup1.pdf]

STRUCTURAL BIOLOGY

Volume 80 (2024)

Supporting information for article:

Investigation of how gate residues in the main channel affect the catalytic activity of *Scytalidium thermophilum* catalase

Yonca Yuzugullu Karakus, Gunce Goc, Melis Zengin Karatas, Sinem Balci Unver, Briony A. Yorke and Arwen R. Pearson

Table S1 Comparison of gate residues at the entrance of the main channel among large subunit catalases.

PDB ID	Catalase	Organism	Gate residues					
4AUM	CATPO	*Scytalidium thermophilum*	Arg134	Gln185	Ala186	Ala187	Thr188	Glu484
2IUF	PVC	*Penicillium vitale*	Arg115	Gln167	Ala168	Ala169	Thr170	Glu466
1SY7	CAT-1	*Neurospora crassa*	Arg123	Gln195	Ala196	Gln197	Ser198	Glu489
1IPH	HPII	*Escherichia coli*	Arg180	Gln231	Gly232	Gln233	Ser234	Glu530
3EJ6	CAT-3	*Neurospora crassa*	Arg123	Gln205	Ala206	Ala207	Thr208	Glu504

Table S2 Kinetic constants

	**Catalase activity			**Phenol oxidase activity				
	*$K_{M\text{-}app}$ (mM)	k_{cat} (s^{-1})	k_{cat}/*$K_{M\text{-}app}$ (s^{-1} M^{-1})	K_M (mM)	k_{cat} (s^{-1})	k_{cat}/K_M (s^{-1} M^{-1})	R_z	Heme
CATPO	10	20.3 x 10^4	20.3 x 10^6	33	7.3 x 10^3	22 x 10^4	0.8	*d*
E484A	8	9.5 x 10^4	11.9 x 10^6	30	43.5 x 10^3	145 x 10^4	0.8	*b*
E484D	11	19.2 x 10^4	17.5 x 10^6	35	7.4 x 10^3	21 x 10^4	0.8	*d*
E484I	18	21.6 x 10^4	12.0 x 10^6	30	26.5 x 10^3	88 x 10^4	0.5	*b*
	(12)	(11.8 x 10^4)	(9.8 x 10^6)	(40)	(12.5 x 10^3)	(31 x 10^4)		
T188A	11	20.9 x 10^4	19.0 x 10^6	33	6.7 x 10^3	20 x 10^4	0.7	*d*
	(11)	(20.1 x 10^4)	(18.3 x 10^6)	(33)	(5.5 x 10^3)	(17 x 10^4)		
T188D	17	21.2 x 10^4	12.5 x 10^6	25	22.3 x 10^3	89 x 10^4	0.5	*B*
	15	10.9 x 10^4	7.3 x 10^6	(33)	(5.7 x 10^3)	(17 x 10^4)		
T188F	6	10.5 x 10^4	17.5 x 10^6	30	30.6 x 10^3	102 x 10^4	0.8	*B*
T188I	21	24.2 x 10^4	11.5 x 10^6	31	28.4 x 10^3	92 x 10^4	0.4	*B*
	(19)	(11.9 x 10^4)	(6.3 x 10^6)	(40)	(8.2 x 10^3)	(20 x 10^4)		

$R_Z = A_{407}/A_{280}$. The values were normalized to heme content in each CATPO variant. The values in parentheses are for unnormalized values and shaded in grey for ease of viewing. *$K_{M\text{-}app}$ is the H_2O_2 concentration at $V_{max}/2$ and is used because the catalase reaction does not saturate with substrate and therefore does not precisely follow Michaelis–Menten kinetics (Switala & Loewen, 2002).

**One unit of catalase is defined as the amount that catalyses the degradation of 1 μmol H_2O_2 in 1 min. One unit of phenol oxidase corresponds to the enzyme catalysing the formation of 1 nmol of product per minute.

Table S3 *B* factors (Å^2) of the water molecules in the main channel of CATPO subunits.

Water #	Subunit A	Subunit B	Subunit C	Subunit D
**WT CATPO (1.4 Å; 4AUM) average *B* factor: 14.02 (all atoms)**				
W1	10.9	11.5	10.0	11.6
W2	16.3	16.8	19.5	20.1
W3	19.0	26.5	18.4	16.2
W4	6.4	8.8	7.3	7.8
W5	6.9	8.5	7.6	8.0
W6	10.0	10.2	8.9	9.4
W7	22.1	22.1	21.7	27.2
W8	26.8	35.5	19.8	27.5
W9	22.8	27.8	23.6	23.8
W10	20.1	19.2	18.6	23.8
W11	25.8	27.5	25.7	18.5
**E484A (1.78 Å; 7WCA) average *B* factor: 26.5 (all atoms)**				
W1	---	---	---	---
W2	19.0	17.9	18.8	17.3
W3	---	---	---	---
W4	16.2	12.5	26.0	13.8
W5	17.0	13.5	13.3	15.6
W6	---	---	---	---
W7	---	---	---	---
W8	---	---	---	---
W9	---	---	34.3	---
W10	---	---	---	---
W11	19.7	25.8	21.8	23.3
**WX**	24.0	25.1	22.6	23.5
**T188A (1.40 Å; 7VN0) average *B* factor: 14.06 (all atoms)**				
W1	30.1	35.0	26.0	31.5
W2	16.1	16.1	15.5	15.6
W3	---	---	44.9	43.2
W4	8.9	7.9	7.0	8.1
W5	11.1	8.4	7.5	9.2
W6	32.6	31.1	29.7	31.3
W7	27.6	29.6	27.5	29.5
W8	27.2	31.8	27.5	---
W9	---	---	---	26.7
W10	---	---	---	---

W11	17.5	21.9	21.3	---
**T188F (1.49 Å; 5YEM) average *B* factor: 13.48 (all atoms)**				
W1	30.3	36.5	32.3	32.0
W2	13.5	13.2	13.0	12.9
W3	52.9	---	---	---
W4	9.9	9.4	9.0	9.7
W5	11.5	10.5	9.5	10.1
W6	28.4	24.4	23.7	25.2
W7	25.6	24.0	21.9	---
W8	27.7	30.6	---	---
W9	---	---	29.6	---
W10	---	---	---	---
W11	---	28.0	19.3	---

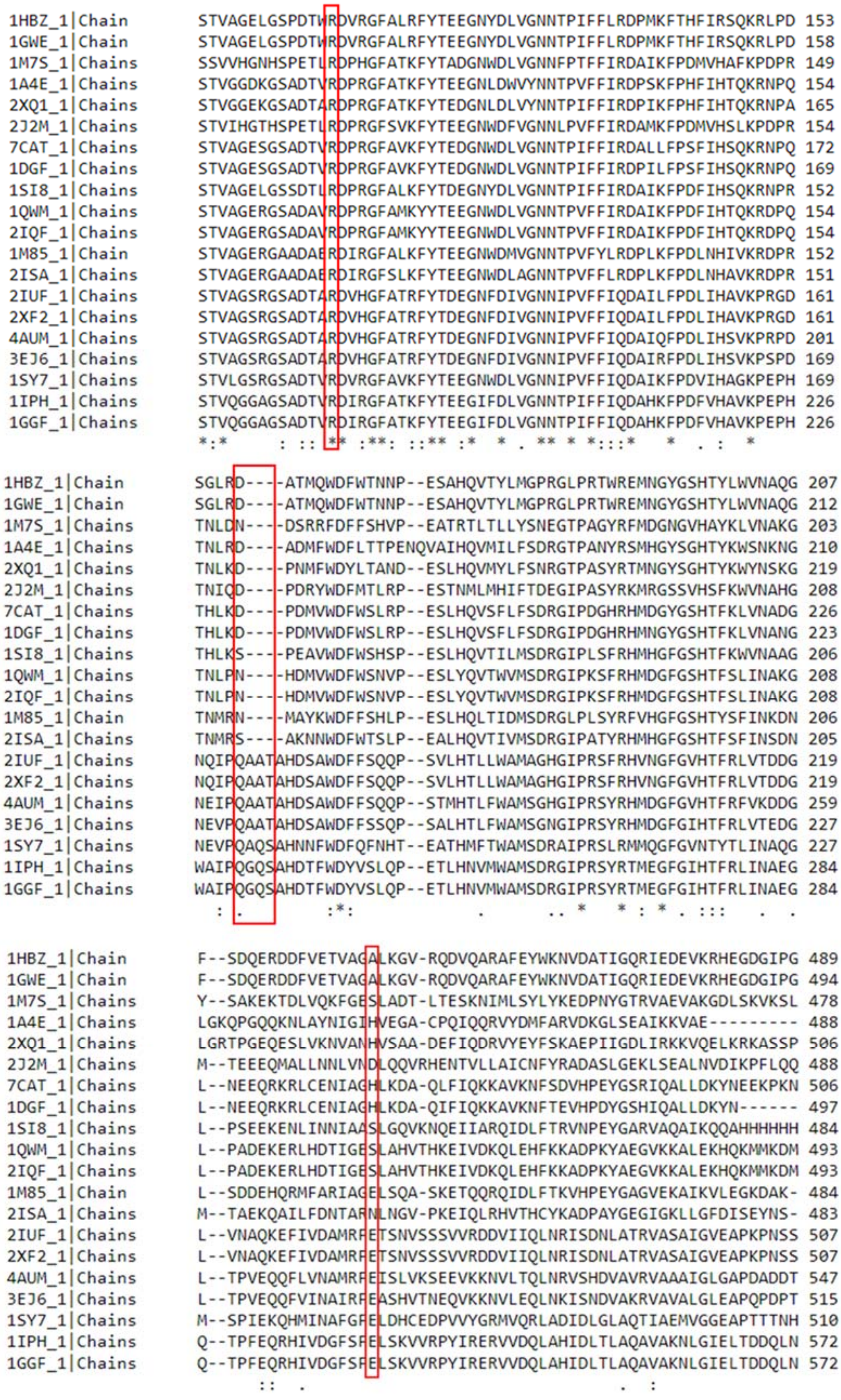
1HBZ_1|Chain STVAGELGSPDTWRDVRGFALRFYTEEGNYDLVGNNTPIFFLRDPMKFTHFIRSQKRLPD 153
1GWE_1|Chain STVAGELGSPDTWRDVRGFALRFYTEEGNYDLVGNNTPIFFLRDPMKFTHFIRSQKRLPD 158
1M7S_1|Chains SSVVHGNHSPETLRDPHGFATKFYTADGNWDLVGNNFPTFFIRDAIKFPDMVHAFKPDPR 149
1A4E_1|Chains STVGGDKGSADTVRDPRGFATKFYTEEGNLDWVYNNTPVFFIRDPSKFPHFIHTQKRNPQ 154
2XQ1_1|Chains STVGGEKGSADTARDPRGFATKFYTEDGNLDLVYNNTPIFFIRDPIKFPHFIHTQKRNPA 165
2J2M_1|Chains STVIHGTHSPETLRDPRGFSVKFYTEEGNWDFVGNNLPVFFIRDAMKFPDMVHSLKPDPR 154
7CAT_1|Chains STVAGESGSADTVRDPRGFAVKFYTEDGNWDLVGNNTPIFFIRDALLFPSFIHSQKRNPQ 172
1DGF_1|Chains STVAGESGSADTVRDPRGFAVKFYTEDGNWDLVGNNTPIFFIRDPILFPSFIHSQKRNPQ 169
1SI8_1|Chains STVAGELGSSDTLRDPRGFALKFYTDEGNYDLVGNNTPIFFIRDAIKFPDFIHSQKRNPR 152
1QWM_1|Chains STVAGERGSADAVRDPRGFAMKYYTEEGNWDLVGNNTPVFFIRDAIKFPDFIHTQKRDPQ 154
2IQF_1|Chains STVAGERGSADAVRDPRGFAMKYYTEEGNWDLVGNNTPVFFIRDAIKFPDFIHTQKRDPQ 154
1M85_1|Chain STVAGERGAADAERDIRGFALKFYTEEGNWDMVGNNTPVFYLRDPLKFPDLNHIVKRDPR 152
2ISA_1|Chains STVAGERGAADAERDIRGFSLKFYTEEGNWDLAGNNTPVFFLRDPLKFPDLNHAVKRDPR 151
2IUF_1|Chains STVAGSRGSADTARDVHGFATRFYTDEGNFDIVGNNIPVFFIQDAILFPDLIHAVKPRGD 161
2XF2_1|Chains STVAGSRGSADTARDVHGFATRFYTDEGNFDIVGNNIPVFFIQDAILFPDLIHAVKPRGD 161
4AUM_1|Chains STVAGSRGSADTARDVHGFATRFYTDEGNFDIVGNNIPVFFIQDAIQFPDLIHSVKPRPD 201
3EJ6_1|Chains STVAGSRGSADTARDVHGFATRFYTDEGNFDIVGNNIPVFFIQDAIRFPDLIHSVKPSPD 169
1SY7_1|Chains STVLGSRGSADTVRDVRGFAVKFYTEEGNWDLVGNNIPVFFIQDAIKFPDVIHAGKPEPH 169
1IPH_1|Chains STVQGGAGSADTVRDIRGFATKFYTEEGIFDLVGNNTPIFFIQDAHKFPDFVHAVKPEPH 226
1GGF_1|Chains STVQGGAGSADTVRDIRGFATKFYTEEGIFDLVGNNTPIFFIQDAHKFPDFVHAVKPEPH 226
1HBZ_1|Chain SGLRD----ATMQWDFWTNNP--ESAHQVTYLMGPRGLPRTWREMNGYGSHTYLWVNAQG 207
1GWE_1|Chain SGLRD----ATMQWDFWTNNP--ESAHQVTYLMGPRGLPRTWREMNGYGSHTYLWVNAQG 212
1M7S_1|Chains TNLDN----DSRRFDFFSHVP--EATRTLTLLYSNEGTPAGYRFMDGNGVHAYKLVNAKG 203
1A4E_1|Chains TNLRD----ADMFWDFLTTPENQVAIHQVMILFSDRGTPANYRSMHGYSGHTYKWSNKNG 210
2XQ1_1|Chains TNLKD----PNMFWDYLTAND--ESLHQVMYLFSNRGTPASYRTMNGYSGHTYKWYNSKG 219
2J2M_1|Chains TNIQD----PDRYWDFMTLRP--ESTNMLMHIFTDEGIPASYRKMRGSSVHSFKWVNAHG 208
7CAT_1|Chains THLKD----PDMVWDFWSLRP--ESLHQVSFLFSDRGIPDGHRHMDGYGSHTFKLVNADG 226
1DGF_1|Chains THLKD----PDMVWDFWSLRP--ESLHQVSFLFSDRGIPDGHRHMNGYGSHTFKLVNANG 223
1SI8_1|Chains THLKS----PEAVWDFWSHSP--ESLHQVTILMSDRGIPLSFRHMHGFGSHTFKWVNAAG 206
1QWM_1|Chains TNLPN----HDMVWDFWSNVP--ESLYQVTWVMSDRGIPKSFRHMDGFGSHTFSLINAKG 208
2IQF_1|Chains TNLPN----HDMVWDFWSNVP--ESLYQVTWVMSDRGIPKSFRHMDGFGSHTFSLINAKG 208
1M85_1|Chain TNMRN----MAYKWDFFSHLP--ESLHQLTIDMSDRGLPLSYRFVHGFGSHTYSFINKDN 206
2ISA_1|Chains TNMRS----AKNNWDFWTSLP--EALHQVTIVMSDRGIPATYRHMHGFGSHTFSFINSDN 205
2IUF_1|Chains NQIPQAATAHDSAWDFFSQQP--SVLHTLLWAMAGHGIPRSFRHVNGFGVHTFRLVTDDG 219
2XF2_1|Chains NQIPQAATAHDSAWDFFSQQP--SVLHTLLWAMAGHGIPRSFRHVNGFGVHTFRLVTDDG 219
4AUM_1|Chains NEIPQAATAHDSAWDFFSQQP--STMHTLFWAMSGHGIPRSYRHMDGFGVHTFRFVKDDG 259
3EJ6_1|Chains NEVPQAATAHDSAWDFFSSQP--SALHTLFWAMSGNGIPRSYRHMDGFGIHTFRLVTEDG 227
1SY7_1|Chains NEVPQAQSAHNNFWDFQFNHT--EATHMFTWAMSDRAIPRSLRMMQGFGVNTYTLINAQG 227
1IPH_1|Chains WAIPQGQSAHDTFWDYVSLQP--ETLHNVMWAMSDRGIPRSYRTMEGFGIHTFRLINAEG 284
1GGF_1|Chains WAIPQGQSAHDTFWDYVSLQP--ETLHNVMWAMSDRGIPRSYRTMEGFGIHTFRLINAEG 284
1HBZ_1|Chain F--SDQERDDFVETVAGALKGV-RQDVQARAFEYWKNVDATIGQRIEDEVKRHEGDGIPG 489
1GWE_1|Chain F--SDQERDDFVETVAGALKGV-RQDVQARAFEYWKNVDATIGQRIEDEVKRHEGDGIPG 494
1M7S_1|Chains Y--SAKEKTDLVQKFGESLADT-LTESKNIMLSYLYKEDPNYGTRVAEVAKGDLSKVKSL 478
1A4E_1|Chains LGKQPGQQKNLAYNIGIHVEGA-CPQIQQRVYDMFARVDKGLSEAIKKVAE--------- 488
2XQ1_1|Chains LGRTPGEQESLVKNVANHVSAA-DEFIQDRVYEYFSKAEPIIGDLIRKKVQELKRKASSP 506
2J2M_1|Chains M--TEEEQMALLNNLVNDLQQVRHENTVLLAICNFYRADASLGEKLSEALNVDIKPFLQQ 488
7CAT_1|Chains L--NEEQRKRLCENIAGHLKDA-QLFIQKKAVKNFSDVHPEYGSRIQALLDKYNEEKPKN 506
1DGF_1|Chains L--NEEQRKRLCENIAGHLKDA-QIFIQKKAVKNFTEVHPDYGSHIQALLDKYN------ 497
1SI8_1|Chains L--PSEEKENLINNIAASLGQVKNQEIIARQIDLFTRVNPEYGARVAQAIKQQAHHHHHH 484
1QWM_1|Chains L--PADEKERLHDTIGESLAHVTHKEIVDKQLEHFKKADPKYAEGVKKALEKHQKMMKDM 493
2IQF_1|Chains L--PADEKERLHDTIGESLAHVTHKEIVDKQLEHFKKADPKYAEGVKKALEKHQKMMKDM 493
1M85_1|Chain L--SDDEHQRMFARIAGELSQA-SKETQQRQIDLFTKVHPEYGAGVEKAIKVLEGKDAK- 484
2ISA_1|Chains M--TAEKQAILFDNTARNLNGV-PKEIQLRHVTHCYKADPAYGEGIGKLLGFDISEYNS- 483
2IUF_1|Chains L--VNAQKEFIVDAMRFETSNVSSSVVRDDVIIQLNRISDNLATRVASAIGVEAPKPNSS 507
2XF2_1|Chains L--VNAQKEFIVDAMRFETSNVSSSVVRDDVIIQLNRISDNLATRVASAIGVEAPKPNSS 507
4AUM_1|Chains L--TPVEQQFLVNAMRFEISLVKSEEVKKNVLTQLNRVSHDVAVRVAAAIGLGAPDADDT 547
3EJ6_1|Chains L--TPVEQQFVINAIRFEASHVTNEQVKKNVLEQLNKISNDVAKRVAVALGLEAPQPDPT 515
1SY7_1|Chains M--SPIEKQHMINAFGFELDHCEDPVVYGRMVQRLADIDLGLAQTIAEMVGGEAPTTTNH 510
1IPH_1|Chains Q--TPFEQRHIVDGFSFELSKVVRPYIRERVVDQLAHIDLTLAQAVAKNLGIELTDDQLN 572
1GGF_1|Chains Q--TPFEQRHIVDGFSFELSKVVRPYIRERVVDQLAHIDLTLAQAVAKNLGIELTDDQLN 572

Figure S1 Multiple sequence alignment of CATPO (PDB no: 4AUM) from *S. thermophilum* with mono-functional heme catalases from eukaryotic and bacterial species. Alignment was performed by using PDB numbers, 7CAT: Beef liver catalase (BLC), 1IPH: Catalase HPII from *Escherichia coli*, 1A4E: Catalase A from *Saccharomyces cerevisiae*; 1DGF: Human erythrocyte catalase, 1GGF: Variant of catalase HPII from *Escherichia coli*, 1HBZ: Catalase from *Micrococcus lysodeikticus* (*M. luteus*), 1GWE: *Micrococcus lysodeikticus* catalase; 1M7S: Catalase CatF of *Pseudomonas syringae*; 1M85: *Proteus mirabilis* catalase; 1QWM: *Helicobacter pylori* catalase with formic acid bound; 1SI8: *Enterococcus faecalis* catalase; 1SY7: Catalase-1 from *Neurospora crassa*; 2IUF: *Penicillium vitale* catalase; 2J2M: Catalase from *Exiguobacterium oxidotolerans*; 2ISA: *Vibrio salmonicida* catalase; 2IQF: *H. pylori* catalase compound I; 3EJ6: *N. crassa* Catalase-3; 2XF2: *P. janthinellum* catalase-ATR (Aminotriazole); 2XQ1: Peroxisomal catalase from the yeast *Hansenula polymorpha.* The gate residues R134, Q185, A186, A187, T188, and E484 in CATPO and its corresponding residues in other catalases are shown in a red frame.

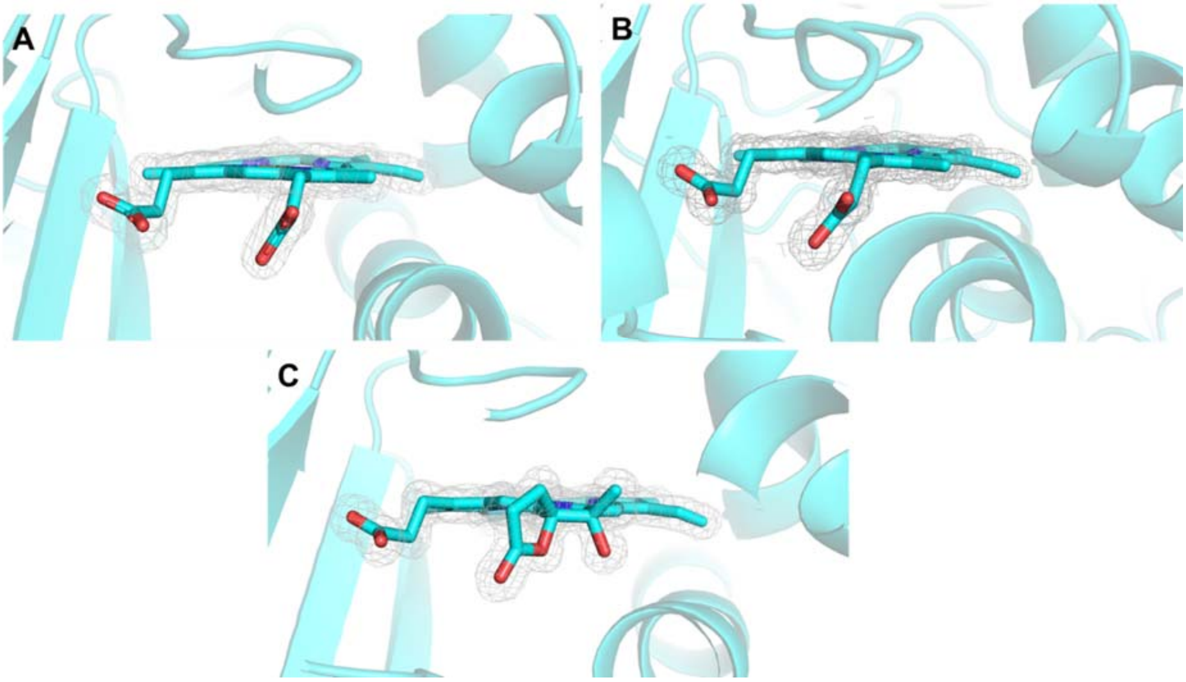

Figure S2 2mFo-dFc Electron density maps (shown in blue at 1.0 r.m.s.d.) of heme *b* in E484A (A) and T188F (B) and heme *d* in T188A (C).

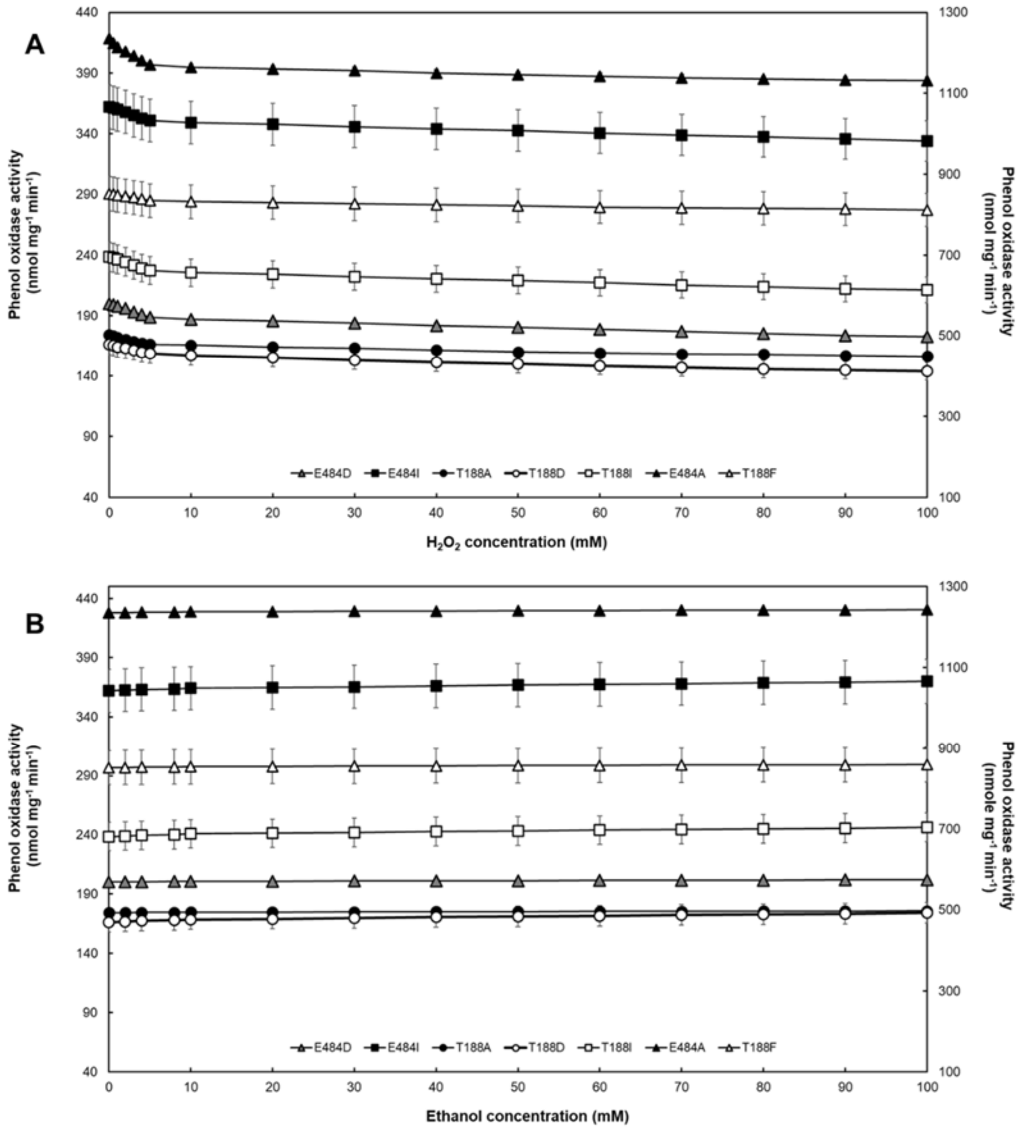

Figure S3 Effect of H_2O_2 (A) and ethanol (B) on phenol oxidase activities of the E484A, E484D, E484I, T188A, T188D, T188F and T188I variants. The enzyme activity was performed under standard assay conditions. The values are presented as means ±SD.

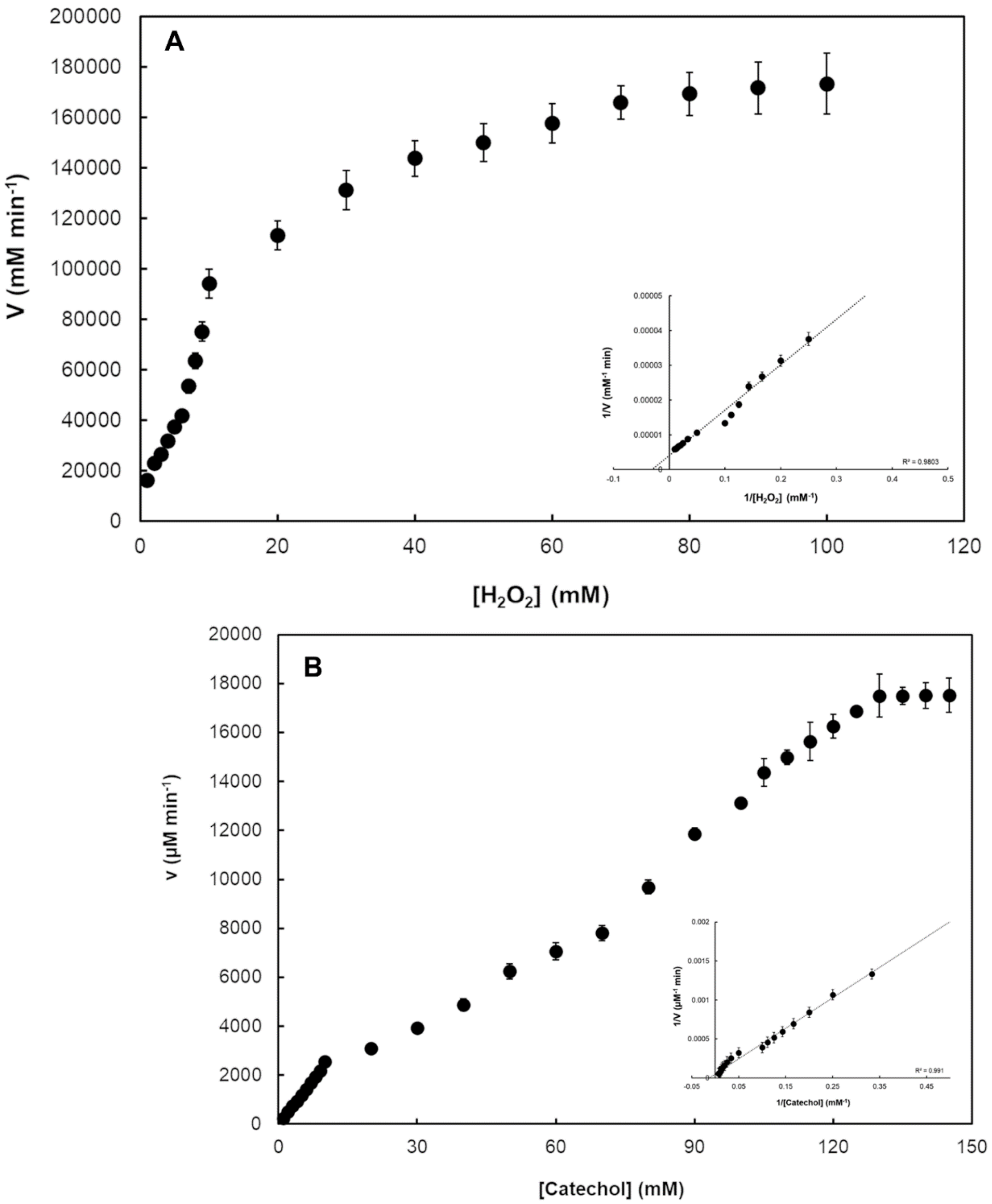

Figure S4 Dependence of wild type enzyme velocity on H_2O_2 concentration (catalase reaction in A) or catechol concentration (oxidase reaction in B). A non-saturated model is given here because catalases never reach the Michaelis-Menten V_{max} predicted by extrapolation from rates catalase activity can't be saturated, so the V_{max} is only ever an estimate (Switala & Loewen, 2002). Insets show double-reciprocal plots of curves in the same panels. Error bars show the standard deviation of the SigmaPlot fit of the raw data for each point.

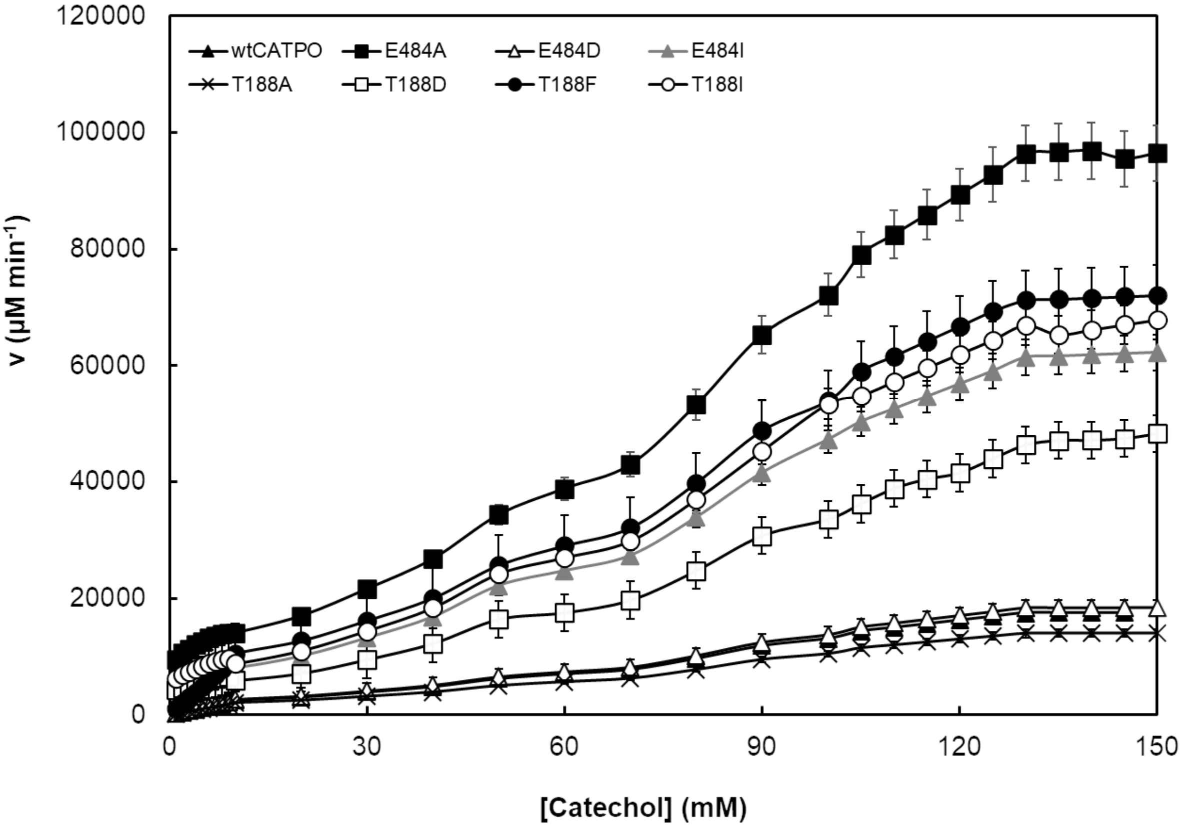

Figure S5 Comparison of the catechol oxidation profile between wild type CATPO and its variants. Lines are provided as a guide to the eye and do not represent fits of a kinetic model.

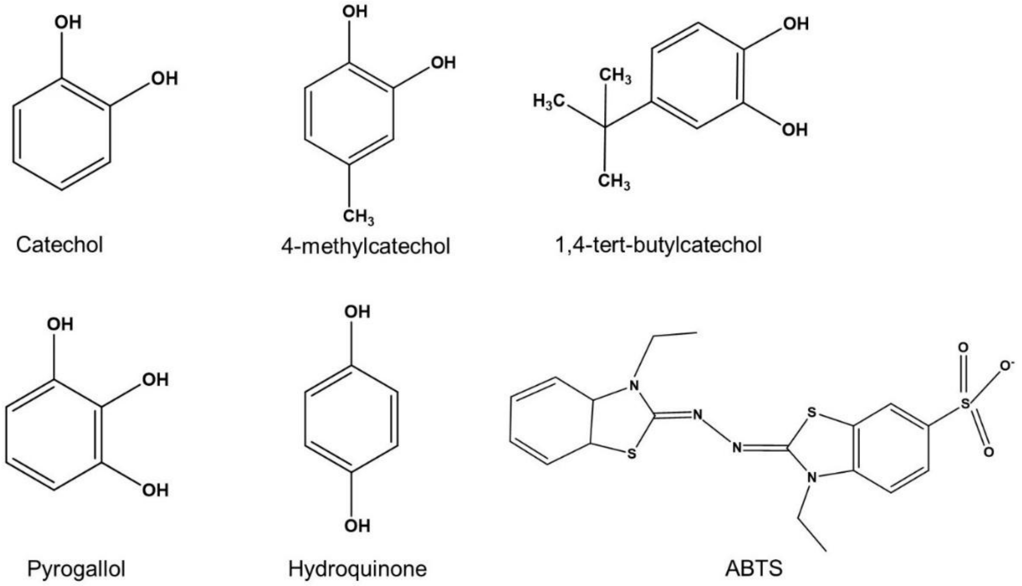

Figure S6 Structures of common polyphenol oxidase substrates used in this study.

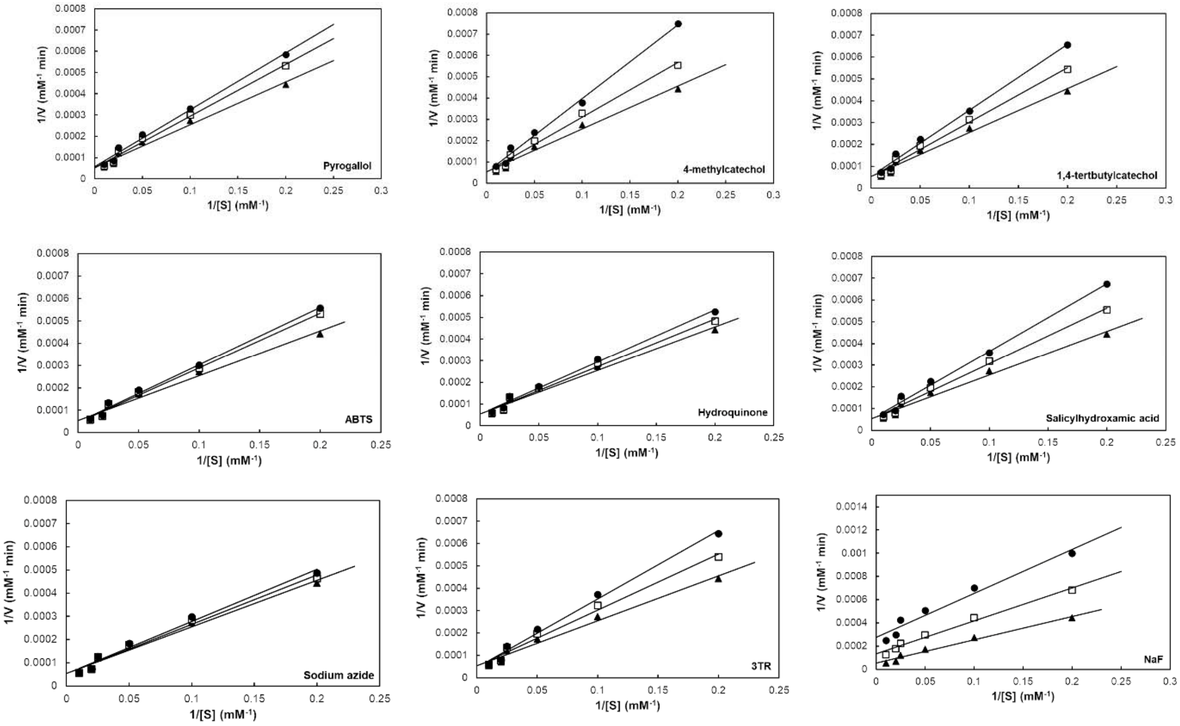

Figure S7 Inhibition kinetics of catechol oxidation by wild type CATPO. Oxidation of catechol by CATPO enzyme was quantified in standard reaction mixes in the absence (closed triangle) and presence of 0.1 mM (open squares) or 10 mM (circles) NaF, or 1 mM (open squares) or 5 mM (circles) 4-methylcatechol, 1,4-tertbutylcatechol and salicylhydroxamic acid or 5 mM (open squares) or 50 mM (circles) ABTS, sodium azide and 3TR.

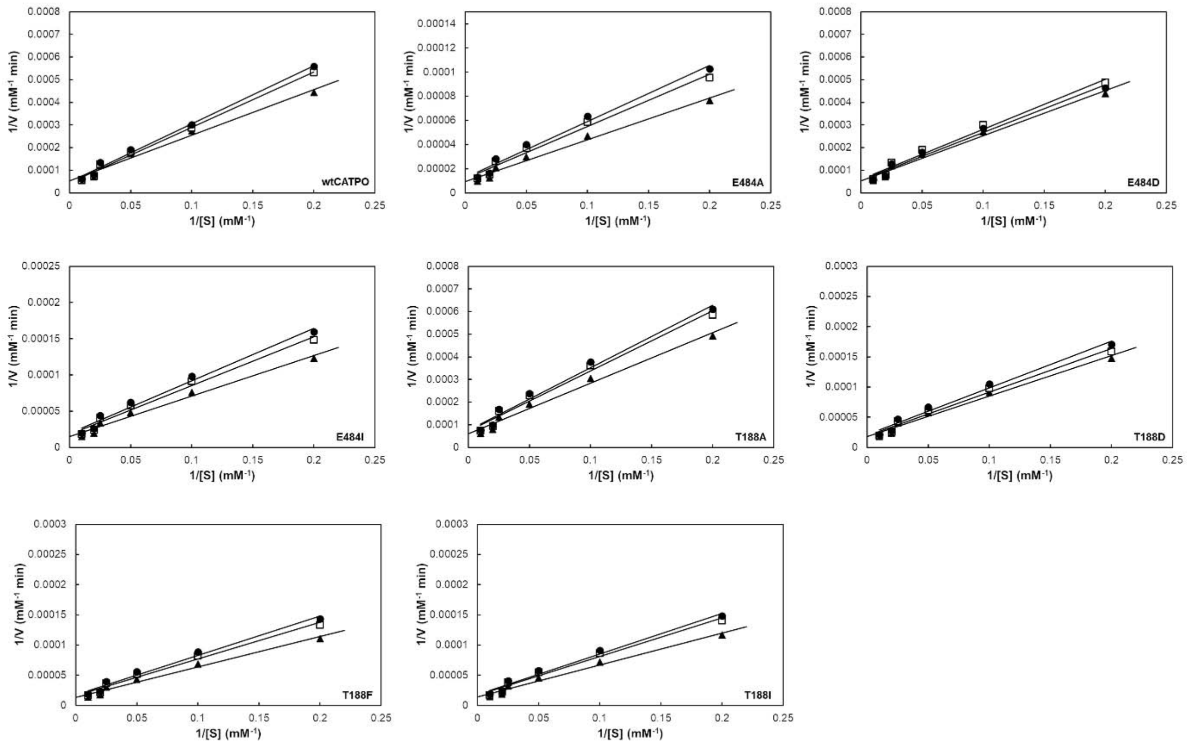

Figure S8 Comparison of inhibition kinetics of catechol oxidation by wild type CATPO and its variants in the absence (closed triangle) and presence of 5 mM (open squares) or 50 mM (circles) ABTS.

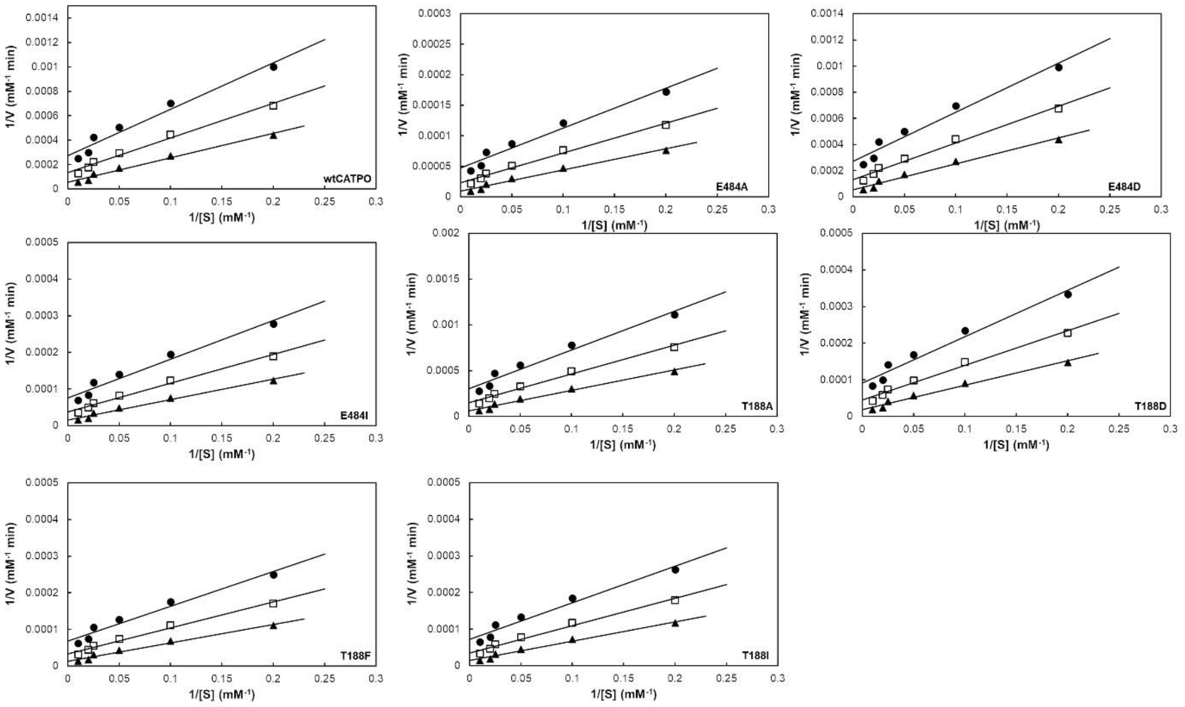

Figure S9 Comparison of inhibition kinetics of catechol oxidation by wild type CATPO and its variants in the absence (closed triangle) and presence of 0.1 mM (open squares) or 10 mM (circles) NaF.

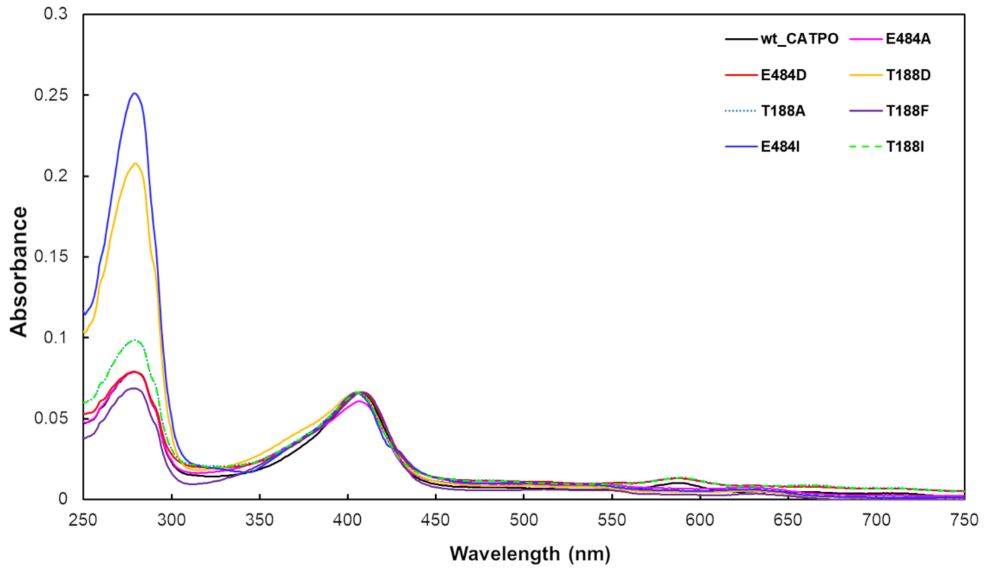

Figure S10 UV-Vis spectra of the CATPO and the E484A, E484I, E484D, T188A, T188D, T188F and T188I variants. The spectra were obtained in 100 mM sodium phosphate pH 7.0 at room temperature. The wild-type CATPO (WT_CATPO) spectrum is adjusted to have an equivalent value at the Soret peak for each of mutants.
